# Effects of early predation and social cues on the relationship between laterality and personality

**DOI:** 10.1093/beheco/arae012

**Published:** 2024-03-06

**Authors:** Paolo Panizzon, Jakob Gismann, Bernd Riedstra, Marion Nicolaus, Culum Brown, Ton Groothuis

**Affiliations:** Department of Biological Sciences, Macquarie University, Wallumattagal Campus, Macquarie Park, Culloden Rd 205 BCR, NSW 2000 Sydney, Australia; Behavioral Biology, Groningen Institute for Evolutionary Life Sciences, University of Groningen, Linnaeusborg (1st floor), Nijenborgh 7, 9747 AG Groningen, The Netherlands; Behavioral Biology, Groningen Institute for Evolutionary Life Sciences, University of Groningen, Linnaeusborg (1st floor), Nijenborgh 7, 9747 AG Groningen, The Netherlands; Behavioral Biology, Groningen Institute for Evolutionary Life Sciences, University of Groningen, Linnaeusborg (1st floor), Nijenborgh 7, 9747 AG Groningen, The Netherlands; Behavioral Biology, Groningen Institute for Evolutionary Life Sciences, University of Groningen, Linnaeusborg (1st floor), Nijenborgh 7, 9747 AG Groningen, The Netherlands; Department of Biological Sciences, Macquarie University, Wallumattagal Campus, Macquarie Park, Culloden Rd 205 BCR, NSW 2000 Sydney, Australia; Behavioral Biology, Groningen Institute for Evolutionary Life Sciences, University of Groningen, Linnaeusborg (1st floor), Nijenborgh 7, 9747 AG Groningen, The Netherlands

**Keywords:** behavioral development, behavioral ecology, laterality, personality, predation

## Abstract

Individual differences in laterality and personality are expected to covary, as emotions are processed differently by the two hemispheres, and personality involves emotional behavior. Fish species are often used to investigate this topic due to the large variability in personality and laterality patterns. While some species show a positive relationship between lateralization strength and boldness, others show a negative relationship, and some show no relationship. A new way to assess the robustness of such a relationship is to manipulate both laterality and personality to examine how this affects their relationship. To this end, we conducted a fully factorial design experiment manipulating predation and group size during early development. Results showed that the strength of laterality was influenced by predation threat, while social tendency and boldness were influenced by group size. These findings suggest that early life conditions can have an impact on laterality and social behavior. The relationship between laterality and personality traits, while present, was heavily influenced by the specific trial conditions but not by the different developmental conditions. In summary, the relationship between laterality and behaviors appears to be context-dependent, yet resilient to early environmental manipulations.

## Teaser

Our latest study shows that laterality and personality are linked, regardless of early environment conditions. By manipulating predation and social environment, we influenced the degree of both laterality and personality, while the correlation between them was not affected. Short-term context seems to be more important in influencing the relationship between laterality and personality than early development.

## INTRODUCTION

Behavioral lateralization is usually attributed to an underlying asymmetry of the brain in processing information and or executing motor patterns (Miletto Petrazzini et al. 2020). While initially believed to be specific to humans (Warren 1980), during the last decade, it has become apparent that behavioral lateralization is a fundamental aspect of the organization of brain and behavior in vertebrates (Wiper 2017). Examples include handedness and facial expressions in humans and primates (Marcori and Okazaki, 2020), paw or leg use in cats, dogs, birds, and amphibians (Ocklenburg et al. 2021), eye preference in birds and fish (Dadda and Bisazza 2006b; Rogers and Kaplan 2006). The body of research on behavioral laterality has increased greatly over the past decade (Ocklenburg et al. 2021), not only to understand its underlying mechanisms but also its ecology and evolution.

Animals have individual internal states that shape their subjective experiences as either pleasant or unpleasant, which can be called emotional states; these include both the long-term affect of an individual, and short-lived reactions directed toward a stimulus (either an object, an environment, or another individual) (Goursot et al. 2021). As a side note, it is worth noting that the subjective experiences of experimental individuals during testing may differ from our own interpretations. It has been proposed that if the processing of environmental stimuli is lateralized, then it should be possible to observe this in the behavior of individuals as a preference to use one side of the body with respect to the other in response to strong emotive stimuli (Leliveld et al. 2013). Such an idea is often referred to as the laterality-valence hypothesis (Rogers 2000). For example, many tetrapod species show a preference to use the left side of the body when interacting with a possibly aggressive or threatening stimulus (Deckel 1995; Austin and Rogers 2012; Leliveld et al. 2013; Siniscalchi et al. 2017, 2021). This suggests that the processing of the emotional reaction to such stimuli is located mainly in the right hemisphere because the right hemisphere controls the left part of the body and vice versa. Since consistent individual differences in behavior are part of animal personality (Carter et al. 2013), and these encompass emotional reactions such as aggression, boldness, and fear, individual variations in laterality and personality are expected to correlate. Personality is defined as intra-population variability in behaviors that are consistent among individuals across time and context (Stamps and Groothuis 2010). Examples include the reaction to risky situations (Burns 2008), the activity level of the individual (Careau et al. 2008), or how they interact with other individuals in social contexts (Bergmüller and Taborsky 2010).

Teleost fish comprise almost half of all vertebrate species (Ravi and Venkatesh 2008). Fish species have been used for laterality research for over 25 years (Bisazza 1996; Cantalupo et al. 1996; Bisazza and Brown 2011; Miletto Petrazzini et al. 2020) and they are also frequently used for personality research (Brown et al. 2005; Bell and Sih 2007; Dingemanse et al. 2007; Budaev and Brown 2011; Conrad et al. 2011; Polverino et al. 2016). Fish are considered a good model for laterality research because their brains have very little cross-communication between the hemispheres and most have laterally placed eyes with little overlap in the frontal visual field, so any behavioral bias reflects processing stimuli in a specific hemisphere, especially regarding visual stimuli (Miletto Petrazzini et al. 2020). The pattern of lateralization in fish varies depending on the species (Bisazza et al. 2000), the population within the same species (Brown et al. 2004), and the individual, often with no preferred side bias at the population level (Brown and Bibost 2014). Such variability in laterality within populations might facilitate correlative analyses with variation in personality.

When studying the relationship between personality and lateralization of behavioral reactions, fish show different patterns depending on the species. For instance, a study in convict cichlids (*Archocentrus nigrofasciatus*) found that the strength of the eye choice while looking at a familiar environment was negatively correlated with the time to emerge from a shelter, a common measure of boldness, suggesting a positive relationship between the strength of visual lateralization and boldness in this species (Reddon and Hurd 2009). In contrast, in the black-lined rainbowfish (*Melanotaenia nigrans*), Brown and Bibost (2014) found that more strongly lateralized fish were less bold. In addition, right-lateralized fish were less bold than left-lateralized ones (Brown and Bibost 2014). In a feral guppy population (*Poecilia reticulata*), an indication of such a negative relationship between boldness and strength of laterality was found to be present only in females (Irving and Brown 2013). More recently, a study in an elasmobranch fish, the Port Jackson shark (*Heterodontus portusjacksoni*), found that strength of laterality negatively correlated with stress reactivity (Byrnes et al. 2016). Finally, a study on an Indian cyprinid (*Tor khudree*) looked for covariation between laterality and either boldness or activity, finding no evidence for a correlation between laterality and either of those personality traits (Varma et al. 2020).

Laterality and most personality traits show plasticity in reaction to environmental cues, thus manipulating the environment to alter the development of laterality and personality is a promising approach to investigate how robust such a relationship between laterality and personality traits is. One of the most studied environmental cues in this regard is predation pressure. Fish living under high predation pressure have stronger behavioral lateralization compared to individuals living under low predation pressure (Brown et al. 2004, 2007). Moreover, by manipulating perceived predation pressure, it was possible to induce a similar effect, both over the course of the development (Broder and Angeloni 2014; Dadda et al. 2020) and as a relatively fast plastic effect over the course of a few days of exposure (Brown et al. 2007; Ferrari et al. 2015, 2017). The most common explanation for this effect regards the ability to split attention between a threat and another stimulus (e.g., food or conspecifics); a more strongly lateralized brain would therefore be more efficient in a predation context (Vallortigara and Rogers 2005) where fish have to pay attention to different stimuli simultaneously, for example, following conspecifics when seeing a predator (Vallortigara and Rogers 2005). It has indeed been shown that, like in the domestic chick (Rogers et al. 2004), lateralized individual fish were indeed more efficient at foraging in the presence of a threat such as a predator (Dadda and Bisazza 2006b) or a harassing conspecific (Dadda and Bisazza 2006a).

The social environment may be another factor that affects the development of laterality. It has been proposed that aligned side biases at the population or species level can arise as an evolutionary stable strategy when such alignment is an important part of a species’ ecology (Brown 2005; Frasnelli and Vallortigara 2018). This theory has found support in theoretical evolutionary models where, in social species, the advantages of being coordinated in swimming direction and/or escape responses outweigh the costs of being predictable for the predator (Ghirlanda and Vallortigara 2004). Many fish species rely on coordinated behavior (schooling) to evade predators (Magurran 1990), suggesting that more gregarious species should show some degree of population (if not species) laterality. Bisazza and colleagues tested this hypothesis using 16 different species of fish and found that schooling species were more likely to be strongly lateralized than non-schooling species (Bisazza et al. 2000). More recently, it has been found that escape responses are more efficient when the members of a school have a similar directional bias; moreover, such an effect was highly context-dependent, being apparent only when the individuals perceived a predation threat in the environment (Chivers et al. 2016).

The aim of this study was to examine how the rearing environment influenced laterality and personality and their relationship in sticklebacks. By manipulating predation (low vs. high perceived predation risk) and the social environment (small vs. large group size) simultaneously in a two-by-two design, we expected to see a positive effect on the strength of laterality induced by enhanced exposure to predatory cues (Broder and Angeloni 2014), and by large group size (Chivers et al. 2016). Since it has been proposed that predator avoidance in groups might be a driver of the evolution of population-level laterality (Dadda and Bisazza 2006b; Chivers et al. 2016; Frasnelli and Vallortigara 2018), we expected to see a stronger effect of the high perceived predation risk and large group size treatments. Laterality was tested in a controlled social context, using a mirror test (Cattelan et al. 2017). Tests for boldness, sociability, and activity were conducted to ascertain personalities. Previous literature suggests that high predation may induce the development of bolder personalities (Meuthen et al. 2019) and a tendency to form larger shoals (Orpwood et al. 2008). If the two hemispheres process strong emotional stimuli differently, we expect to see laterality and personality traits, especially boldness (Reddon and Hurd 2009; Brown and Bibost 2014), to covary regardless of treatment; more precisely, we expect individuals with stronger lateral bias to show less bold behavior (Reddon and Hurd 2009). According to previous research, the direction of population-level lateralized behavior presents high variability between different species of fish (Bisazza and Brown 2011); because of that, and since there is very little laterality research on Three-spined sticklebacks (but see (Jutfelt et al. 2013; McLean and Morrell 2021)), we do not have a prediction for the specific direction of lateral bias.

## MATERIALS AND METHODS

### Study species

This experiment was conducted at the University of Groningen’s animal facility using native three-spined sticklebacks (*Gasterosteus aculeatus*). The individuals used in this experiment hatched between May and June 2020. They were direct offspring of wild sticklebacks caught in April 2020 at the Ems-Dollard Estuary, on the northern Dutch-German border and reared in a semi-natural environment (Ramesh et al. 2021). These individuals were used as breeding population and their offspring’s rearing environment was manipulated. In the wild, parents and offspring often experience similar environments, so here, we exposed both parents and offspring to either predation cues or no such cues (see below for details). Perceived predation in the environment is known to affect the offspring’s behavior via parental effects (Mommer and Bell 2013; Cattelan et al. 2020). In the offspring, group size was also manipulated to affect the social environment, again as described below. We did not manipulate group size in the adults, since this would have influenced the genetic variability of the offspring. This species and the pond system were chosen for three reasons. First, as a study species, the three-spined stickleback is a widely used teleost fish in behavioral ecology research, and much is known about its ecology, behavior, and development (Huntingford and Ruiz-Gomez 2009), while very little research has been conducted on laterality (but see (McLean and Morrell 2021)). Second, the development of laterality in fish seems to be impaired by impoverished rearing environments (Berlinghieri et al. 2021), making the choice of a semi-natural, enriched condition preferable. Lastly, this population and the methodology we used had already proved successful for personality research (Ramesh et al. 2021).

### Treatment

#### Breeding

One hundred individuals (50 males and 50 females) were housed in the ponds during late spring of 2021, the common breeding season for sticklebacks (Östlund-Nilsson et al. 2007). The females’ size ranged between 40 and 45 mm. The fish were equally divided into two sets of five semi-natural, circular ponds (Volume ~1000 L). The ponds in each set were connected to each other by tubes and contained a sandy floor and hiding spots and nesting material (pieces of green sewing thread). Water temperature was monitored to check for extremes outside the average, natural range occurring in the Netherlands during spring (between 10 °C overnight and 20 °C during the day). Ph was checked to be stable at 8. Such setup, conditions, and densities (on average five males and five females per pond) had already been shown to allow for natural breeding to occur (Ramesh et al. 2023). For the entire breeding season (from May to July), in one of the two sets of ponds, a predation treatment was performed (see below), so that 25 males and 25 females were exposed to predation cues (P+ individuals), while 25 males and 25 females were used as control in the other set of five ponds (P− individuals). Fish were fed frozen artemia and frozen bloodworms twice a day. The treatment was performed on each pond at the same time and in the same order, and it lasted approximately 5 min. Three different predatory cues were used: 1) approximately 5 L of water was added from a pond housing three European perches (*Perca fluviatilis*), a common predator of Sticklebacks in the wild (Östlund-Nilsson et al. 2007), 2) 5 mL of extract from the skin and muscle of dead conspecifics (see Supplementary Materials for the protocol of extraction) was added to each pond, and 3) chasing the shoal with a model perch predator (20 cm long) for 30 s. The three cues were applied three times per week on random days.

#### Offspring rearing

Two weeks after the start of the treatment (to allow time for the predation treatment to be effective), ponds were checked daily for nests containing eggs (eggs hatch after approximately 3 days, so all eggs were relatively fresh and laid after a substantial time of treatment). Each nest was collected, and the eggs were removed and housed in buckets of 20 L (“nursery”) next to the ponds. The eggs from P+ ponds were kept in identical but separate buckets as P− eggs. The offspring hatched in the nursery were monitored for 3 to 8 weeks, this variation being due to age difference, and fed two times per day with frozen cyclops larvae. Such a monitoring period before starting the group size treatment was deemed necessary because of the naturally high mortality of larval and fry stage. P+ offspring in the nursery received both odor cues (see above) on random days three times per week. Such a setup was chosen to reinforce the predation cues via possible parental effects (Bell and Sih 2007; Mcghee et al. 2012) while simulating the natural occurrence of predators in both parental and offspring environments. After all the hatched individuals were monitored for at least 3 weeks, the two groups were transferred to separate cages consisting of very fine netting (40 × 40 × 60 cm) that were partly submerged in the ponds (Supplementary Figure A). The total volume of water inside each cage was approximately 70 liters. To manipulate the social environment, we created small groups (D−), which consisted of 5 individuals, and large groups (D+), containing 15 individuals for both P+ and P− treatments using a two-by-two design. Such numbers were chosen as a balance between multiple necessities: not reaching excessive rearing density in the cages, which is known to affect welfare and behavior (Brockmark et al. 2010; Manley et al. 2014; Berlinghieri et al. 2021); allowing for a certain amount of social interactions in both groups; compensating for the expected mortality in young individuals (see below). The density treatment could not be reinforced with parental treatment as well because of the eventual interaction of density on natural breeding. Within treatments, each fish was randomly assigned to a cage and each cage contained hay and stones for simulating a natural environment. In total, we established 4 cages per pond (2 D+ and 2 D−) overall using 12 ponds, (6 of which received the predation treatment and 6 were used as control). The total number of fish housed was, therefore, 480. The same predation treatment described above (alarm substance, predator odor, and chasing with a model) was performed for the P+ cages, except that a smaller predator model was used (12 cm long).

**Figure 1:**
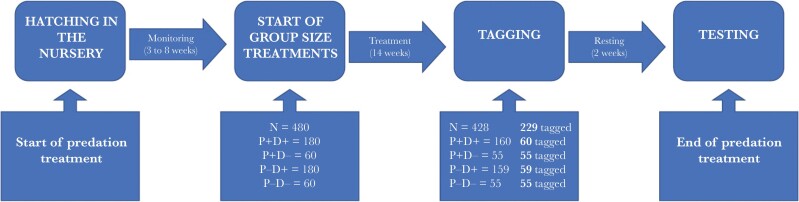
Flow chart of the treatment and rearing process during summer and early autumn. P+D+ refers to large groups with predation treatment, P+D− to small groups with predation treatment, P−D+ are large groups with no predation treatment, P−D− are small groups with no predation treatment.

After 14 weeks of treatment, the fish were moved as a group from the netting cages to one of 48 identical 8-L plastic tanks (29 × 19 × 16 cm), housed in our inside facility for 24 h before being measured and tagged (see Supplementary Materials for the details of the tagging and measuring procedure). Every fish in the small groups were tagged. While tagging the fish in the large groups, a fish was randomly chosen and tagged. If the chosen fish was deemed too small to be tagged (less than 35 mm, see below), the random choice was repeated until a total of 5 fish was tagged. We planned to tag 5 fish per cage (total = 240). A total of 229 fish were tagged, all larger than 35 mm. The final number of tagged fish used in the experiment was lower because of mortality during treatment. A Generalized Linear Mixed Model showed that the different treatment groups had no differences in mortality (see Supplementary Materials). The fish were left in the inside facility for an additional 24 hours to monitor recovery and were then moved back to the cages in the ponds, where they were left undisturbed for two additional weeks to allow complete recovery. The temperature inside was the same as outside, the latter varying over the entire period between 8 and 12 °C. One individual from the P-D+ treatment group did not survive the procedure. See Figure 1 for a scheme of the treatment procedure.

### Testing

Testing started when the fish were between 19 and 24 weeks of age. The testing apparatus consisted of a wooden box (60 × 80 × 80 cm) in which two glass testing tanks (60 × 30 × 30 cm) were fitted (Supplementary Figure B). Each tank was covered on the inside with opaque plastic to prevent the fish from seeing through the walls of the tank, and the bottom was covered with fine, light-colored sand. The tanks were filled to a depth of 8 cm using tap water and the top of the box was closed to minimize any external interference during the testing of the fish. LED lights were used inside at the top of the lid for a dim and uniform illumination during the testing of the fish. At the top, a camera (Raspberry Pi NoIR Camera Board V2 – 8MP, Raspberry Pi Foundation, UK) was fitted to record the behavior of the fish. Testing started 3 weeks after the fish were tagged over 4 consecutive days. The testing was performed in the same room where the fish were housed (see below) to reduce the discomfort caused by transport. On day 1, the tagged fish were collected from the outdoor cages and put in the inside facility as previously mentioned. On day 2, the Activity and the Social Preference Tests (see below) were performed. On day 3, the fish were left undisturbed. On day 4, the Mirror and Predator Interaction Tests (see below) were performed, after which the fish were then released back into the ponds. Water was refreshed in the tanks between the testing days. The fish were fed only at the end of days 2 and day 4. This procedure was repeated 3 weeks later using the same fish.

#### Activity Test

An open-field test was employed to measure the total distance moved by the individuals in a standardized amount of time (Ramesh et al. 2021). A fish was gently placed into the central compartment of the arena (36 × 30 cm, 8 cm deep water) using a net, and we started recording after 30 s. The total testing time was 5 min. The analysis on the recorded video started at the beginning of the videos and stopped approximately 18 s before the partitions were lifted to begin the Social Preference Test (see below) because the movement of the partitions could affect both the behavior of the fish and the automated software’s ability to recognize the fish. The total distance moved by the fish was calculated.

#### Social Preference Test

The Social Preference test was adapted from previous literature to discriminate between the tendency to prefer to shoal with conspecifics from the general tendency to reach safety in novel, risky environments (Wark et al. 2011; Ramesh et al. 2021). The testing tank was split into three parts, separated by two transparent partitions. The two outer parts were 30 × 12 cm wide, while the inner part was 36 × 30 cm wide (both parts with 8 cm deep water) (Supplementary Figure C). Two removable, black partitions also covered the transparent ones. In the two lateral compartments, we housed untagged surplus fish from one of the D+ cage so that two fish of similar size were in one and the remaining eight fish in the other one. The position of the large or small shoal was randomized between different days of testing. No tagged fish from a D+ cage was tested along with untagged stimuli fish from the same cage. The procedure was as follows: After the Activity Test (see above), the black partitions were remotely lifted to reveal the shoals to the focal fish. At the end of the test, the focal fish was removed and put back in the original plastic tank. Approximately 10% of the water in the testing tank was then refreshed.

The video analysis started on average 90 s after the partitions were lifted, to give the fish time to recover from the disturbance caused by lifting the partitions before the start of the recordings. The analysis of the shoaling choice test was, on average, 13.5 min long. An area next to the large shoal of 1.5 body length of distance from the transparent partition was used by the software to calculate the time spent next to the large shoal.

#### Mirror Test

This test was chosen to test for the laterality of the individuals in a social context (Cattelan et al. 2017). The arena was 30 × 12 cm wide (8 cm deep water), with one mirror on each of the shorter sides (Supplementary Figure D). The fish was gently put into the arena with a net, the lid was closed, and the filming started after 30 s. The total testing time was 5 min. To analyze the mirror test, every 3 s, the videos were stopped, and the following data were recorded: If the fish was interacting with the mirror, with which eye it was facing the mirror, or if neither eye was clearly being preferentially used. To define “interacting with the mirror,” the fish’s eyes had to be within 1.5 body lengths from either mirror, and the fish had to face the mirror at an angle within ± 135°. To define “neither eye was preferentially used,” the fish had to be looking at the mirror within ± 15°. The axis formed by the head being perpendicular to the mirror was used as a reference to determine the angles. For each fish, 100 screens were used to approximate 300 s of interaction with the mirror.

#### Predator Interaction Test

This test was chosen to test boldness in a context related to the predation treatment the individuals experienced. Each test tank was split into three parts. The two outer parts were 30 × 12 cm wide, while the inner part was 36 × 30 cm wide (water in both parts was 8 cm deep). One of them was separated by a transparent partition, and the other one was revealed by removing a black partition. The latter contained a model predator, the former two mirrors on the sides (Supplementary Figure D). The predator model was connected to an air tube that caused it to move slightly. The position of the predator and mirror compartments was randomized between different days of testing. At the beginning of the day of testing, 10 mL of damaged conspecific odor cue (see above) was added to the tank. The total testing time was 15 min. The procedure was as follows: After the mirror test, the black partitions were remotely lifted to reveal the inner part and the model predator to the focal fish. At the end of the test, the focal fish was removed and put back in the original plastic tank. Approximately 10% of the water in the testing tank was then refreshed, and 1 mL of odor cue was added again in the testing tank.

For the analysis of the Predator Interaction test, the videos started 90 s after the partitions were lifted, to give the fish time to recover from the disturbance caused by lifting the partitions. From that time point, the analysis was performed to the end of the video (ca. 13 min). The arena was divided between an area between the mirrors, deemed safe, and an equal area next to the predator, deemed risky. The time spent inside each of those two areas was recorded.

#### Software

The data from the Activity Test, the Social Preference Test, and the Predator Interaction Test were collected using EthovisionXT (Noldus Information Technology company) automated video tracking software. The data from the Mirror Test were collected using BORIS v7.13.6 (Friard and Gamba 2016). The videos were cut using ffmpeg. Thanks to the random alphanumeric ID given to every individual, the operator was blind to the treatment of the fish during the analysis. In addition, by using automated video tracking software, the possibility for a bias in data acquisition was avoided.

#### Statistical analysis

Unless differently specified, the Linear Mixed Models (LMMs) mentioned below all share the same structure for random effects, which is fish ID nested into cage ID nested into pond ID to account for how the fish were reared during treatment.

To test for the effect of treatment on Standard Length, we performed an LMM with a cage nested into pond as random effects and predation and group size and the interaction between them as fixed effects. A similar LMM without the interaction effect was performed to test for the main effect of the two treatments. Since there was a strong effect of the treatment on Standard Length (see Supplementary Materials), we did not include Standard Length as covariate in any further analysis to avoid confounding any effect of treatment on the laterality and personality traits.

In the Mirror Test, we tested the probability of each fish to be skewed toward right or left with a Binomial test. We wanted to check, whether the proportion of fish showing a lateralized bias was higher than the one expected by chance (i.e., false positives). By calculating the p-value for each individual, we controlled for the variation in the number of turns between individuals. Only individuals who interacted with the mirror with either the left or the right eye in at least 10 frames were used in this analysis. We then calculated the proportion of individuals being skewed (*P*-value below 0.05) and compared it with the expected proportion if the individuals turn on the left or right by chance using a Chi-Square test.

We tested for individual consistency in laterality across the two trials with an adjusted repeatability estimated with 1000 parametric bootstrap. The same test was used to test for consistency in the personality traits, in order to identify personality traits in our population (Stoffel et al. 2017).

To avoid losing variance in the data, we used a continuous estimate of laterality called Laterality Index, often used in literature. This index was calculated as (R-L)/(R+L), where R is the number of interactions using the right eye and L with the left eye. The strength of laterality irrespective of its direction is the absolute value of the Laterality Index, and it is called Absolute Laterality. The Absolute Laterality index was transformed using the logit transformation to better match the assumptions of the Linear Models.

We ran an LMM with trial as a fixed effect, and the Laterality Index as a response variable. By testing whether the intercept was significantly different than 0, and by including trial in the model, we checked for any population-level direction of laterality and the possible effect of the two trials on it.

Activity was estimated by using the mean speed of the fish during the Activity Test. It was first normalized by dividing the mean speed measured in mm/s by the Standard length measured in mm of each individual, which transformed it to body length/s. As an estimate for boldness, the proportion of time spent in the safe area of the arena during the Predator Interaction Test was used. Such variable was then called Predator Avoidance. This measure was chosen over the proportion of time spent next to the predator (Boldness) because the data matched the assumptions of the LMMs better. The two variables were highly and negatively correlated (Spearman ρ: −0.929, *P*-value < 0.001). Social Tendency was estimated by using the proportion of time spent next to the large shoal during the Social Preference Test.

Since both personality (Frost et al. 2007) and laterality (Ferrari et al. 2017) have shown to be plastic across relatively short time periods, we wanted also to control for such difference, along with the main question about the effect of treatment on behavior. We used the following model structure to analyze the effect of treatment and trial on Activity, Predator Avoidance, Social Tendency, Laterality Index, and Absolute Laterality, running a different model for each one of those behaviors. We used a Linear Mixed Model where we included all the two-way interactions between predation treatment, group size treatment and trial as fixed effect. We did not include the three-way interaction between predation, group size and trial in the final analysis because it did not improve the fitting of the model (see Supplementary Material) and makes it harder to interpret the two-way interactions. To help with the interpretation of any two-way effect, a post-hoc test was run, and multiple testing was accounted for by false discovery rate correction.

To test whether there was a relationship between laterality and personality traits, we ran a multivariate generalized linear model with Monte Carlo approximation of the posterior, with an uninformative prior. We included individual, cage, and nest ID as random effects, allowing for the analysis of both within- and among-individuals (co)variance. The multivariate model was ran with 100,000 iterations, a warm-up of 20,000 iterations, and a thinning interval of 10 (Supplementary Materials). To test whether treatment or trial influenced the relationship between laterality and personality, we used AIC model selection to distinguish among a set of possible models describing the relationship between personality, laterality, trial, and treatment. We compared LMMs with all the two-way interactions between a personality measure, treatment group, and trial as fixed effect, excluding the interaction between treatment and trial. The laterality traits were used as response variables. We checked that the personality traits did not covary with one another before including them as covariates in the same model (Supplementary Materials). The models resulting from all the possible combinations of two-way interactions were compared using AIC (Supplementary Materials).

Statistical analyses were run on RStudio 2022.07.2 + 576; the package “lme4” was used to run the Linear Mixed Models; “rptR” package was used to run the adjusted repeatability estimates. Post-Hoc pairwise comparisons were tested using “emmeans” R package. The multivariate analysis of laterality and personality (co)variation was performed with the “MCMCglmm package” (Hadfield 2010). It was not possible to successfully analyze some of the videos, leading to missing data for some individuals in some tests. Because of this, each model has a slightly different sample size, as reported in the Results section.

## RESULTS

### Overall laterality in the Mirror Test

**Figure 2:**
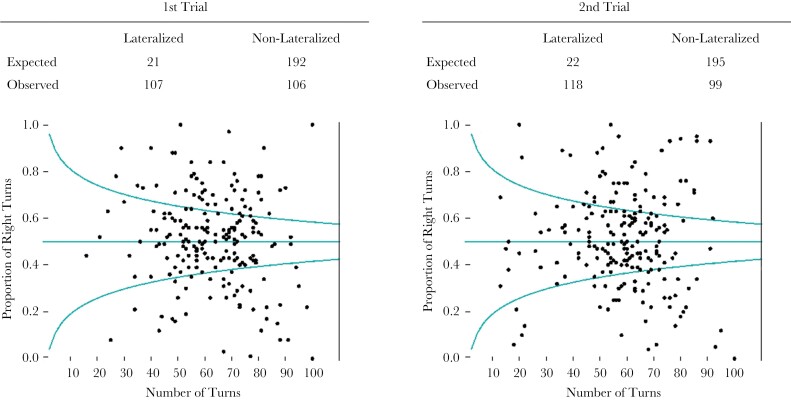
Plotted individual raw data of the proportion of right turns taken vs the total number of turns taken, split by trial. The curves of the 0.05 significance threshold are shown, for both having a skew to the right and to the left. The contingency tables of the expected and observed values are shown. The expected proportion of fish lateralized by chance are calculated as the 10% of the total fish (5% probability for either direction).

The results of the binomial test for the individual fish showed that the number of individuals significantly skewed (either to the left or right) was different from the expected number of random turning choice in both trials (Figure 2) (Chi-Squared tests; first trial: χ^2^ = 82.6, *P* < 0.001; second trial: χ^2^ = 97.2, *P* < 0.001). The fish did not show consistency at the individual level between the two trials (Laterality Index adjusted repeatability estimate: *R* = 0, SE = 0.041, *P*-value = 1; Absolute Laterality adjusted repeatability estimate: *R* = 0, SE = 0.041, *P* = 1). The value of the intercept in the LMM was not different from 0 (estimate: 0.039, SEM: 0.028, *P* = 0.173), and there was no effect of the trial on the average of the Laterality Index (estimate: −0.030, SEM: 0.038, *P* = 0.439). These results combined show that, while the fish show lateralized behavior within the single trials, such behavior changes between the trials, and there was no population-level laterality in either the first, nor the second trial.

### Effect of treatment on laterality

**Figure 3:**
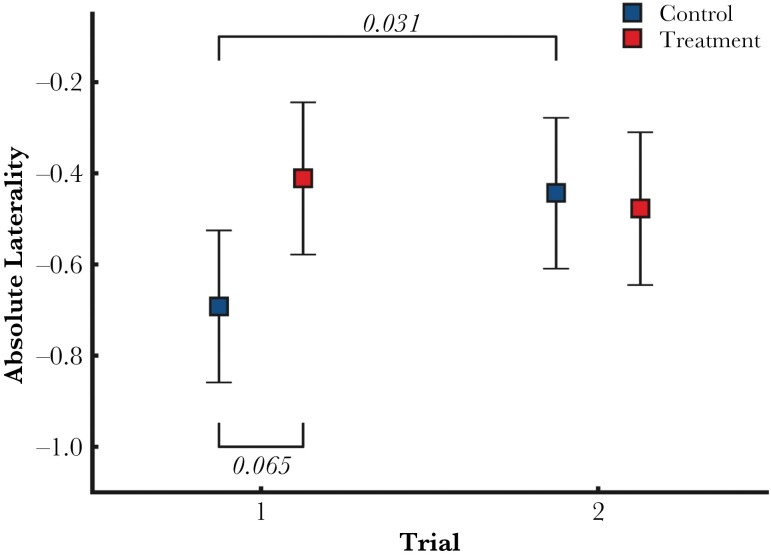
Estimated Marginal Means with 95% CI for the interaction effect of Trial and Predation Treatment of the LMM on absolute laterality. After a pairwise post-hoc test, only the difference between trials within the control group is significant, with absolute laterality increasing on average from the first (95% CI: −0.859, −0.525) to the second trial (95% CI: −0.609, −0.278). A noteworthy, positive effect of predation in the first trial is also shown (Control: 95% CI: −0.859, −0.525; Treatment : 95% CI: −0.578, −0.244).

There were no effects of trial nor treatment on the Laterality Index (Table 1). For the Absolute Laterality scores the result showed an effect of predation and trial and their interaction (Table 1). The Post-Hoc pairwise comparison showed an almost significant tendency for a positive effect of predation in the first trial but not in the second, and the control group significantly increased Absolute Laterality on average between trials (Figure 3).

**Table 1:** Estimates of the LMMs with Laterality as response variable (*N* = 218)

Effect of trial and treatment on laterality								
	Laterality Index					Absolute Laterality		
Predictors	Estimate	CI		*P*		Estimate	CI	*P*
(Intercept)	0.09	−0.02	0.19	0.119	−0.69	−0.88	−0.49	<0.001
Trial	−0.02	−0.16	−0.11	0.728	0.24	0.03	0.46	0.027
Predation	−0.01	−0.16	0.13	0.843	0.33	0.07	0.59	0.013
Group size	−0.10	−0.24	0.04	0.160	−0.01	−0.24	0.21	0.923
Trial × Predation	−0.07	−0.22	0.08	0.339	−0.32	−0.56	−0.07	0.012
Trial × Group size	0.06	−0.09	0.21	0.451	0.01	−0.24	0.26	0.932
Predation × Group size	0.05	−0.12	0.21	0.568	−0.10	−0.37	0.17	0.454

Random effects	Estimated variance		95% CI		Estimated variance		95% CI	
Fish ID	<0.01		<0.01	0.01	<0.01		<0.01	0.06
Cage ID	<0.01		<0.01	0.01	0.01		<0.01	0.04
Pond ID	<0.01		<0.01	<0.01	0.01		<0.01	0.04

### Activity

There were no effects of treatments and trial or their interactions on Activity (Table 2). The estimated adjusted repeatability at the individual level was significantly different from 0 (adjusted repeatability estimate = 0.301, SE = 0.067, *P*-value < 0.001), suggesting Activity scores were moderately repeatable compared to commonly found repeatability scores for activity (Rohrer and Ferkin 2020).

**Table 2:** Estimates of the LMMs with Personality Traits as response variable. *N* = 223 (Activity), *N* = 229 (Social Tendency), *N* = 222 (Predator Avoidance).

Effect of trial and treatment on personality traits												
	Activity				Social tendency				Predator avoidance			
Predictors	Estimate	CI		*P*	Estimate	CI		*P*	Estimate	CI		*P*
(Intercept)	0.07	0.05	0.08	<0.001	0.65	0.59	0.70	<0.001	0.65	0.58	0.72	<0.001
Trial	−0.00	−0.01	0.01	0.471	0.02	−0.04	0.08	0.459	0.01	−0.06	0.08	0.738
Predation	−0.01	−0.02	0.01	0.341	−0.01	−0.09	0.06	0.685	−0.01	−0.10	0.08	0.768
Group size	0.01	−0.00	0.02	0.157	0.06	−0.01	0.12	0.077	−0.10	−0.19	−0.02	0.021
Trial × Predation	−0.00	−0.01	0.01	0.810	−0.03	−0.09	0.04	0.437	−0.06	−0.15	0.02	0.149
Trial × Group size	−0.01	−0.02	0.00	0.203	−0.09	−0.16	−0.03	0.005	−0.00	−0.08	0.08	0.988
Predation × Group size	−0.01	−0.02	0.01	0.423	−0.03	−0.11	0.05	0.462	0.08	−0.02	0.19	0.110

Random effects	Estimated variance		95% CI		Estimated variance		95% CI		Estimated variance		95% CI	
Fish ID	<0.01		<0.01	<0.01	<0.01		<0.01	0.01	<0.01		<0.01	0.01
Cage ID	<0.01		<0.01	<0.01	<0.01		<0.01	<0.01	<0.01		<0.01	0.01
Pond ID	<0.01		<0.01	<0.01	<0.01		<0.01	<0.01	<0.01		<0.01	<0.01

### Social Tendency

**Figure 4:**
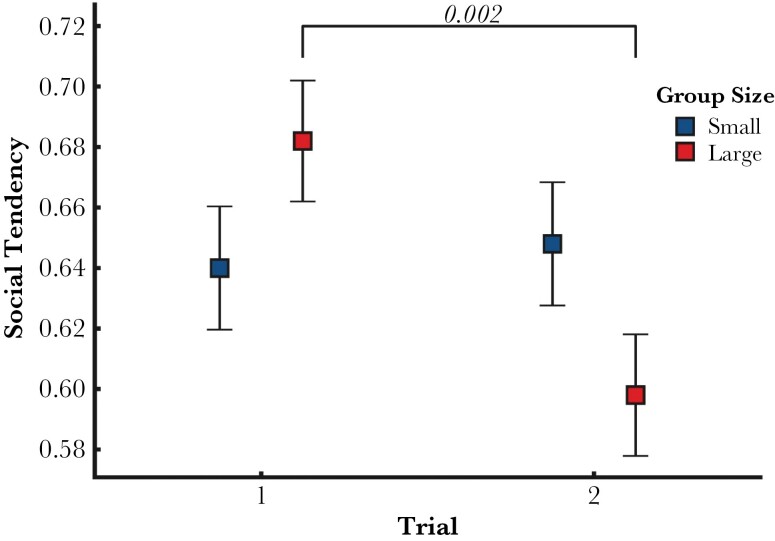
Estimated Marginal Means with 95% C.I. for the interaction effect of Trial and Group Size of the LMM on Social Tendency. After a pairwise post-hoc test, only the difference between trials within the Large Group treatments is significant, with Social Tendency decreasing on average from the first (95% CI: 0.642, 0.722) to the second trial (95% CI: 0.557, 0.638).

There was an interaction effect of trial and group size on the time spent near the large shoal during the Social Tendency test (Table 2). A post-hoc pairwise comparison showed that the tendency to swim next to the large shoal decreased between the first and second trial, but only among the large group treatments, while the small groups did not change across trials (Figure 4). The estimated adjusted repeatability at the individual level was not significantly different from 0 (adjusted repeatability estimate = 0.046, SE = 0.054, *P*-value = 0.245).

### Predator Avoidance

There was a negative effect of group size on Predator Avoidance. A post-hoc *t*-test showed that the fish from the large group size treatment approached the predator more during the test on average (estimate = 0.059, SEM = 0.026, *P*-value = 0.033). No interaction effect was found (Table 2). The estimated adjusted repeatability at the individual level was not significantly different from 0 (adjusted repeatability estimate = 0.066, SE = 0.063, *P*-value = 0.198).

### Covariance of laterality and personality

**Figure 5:**
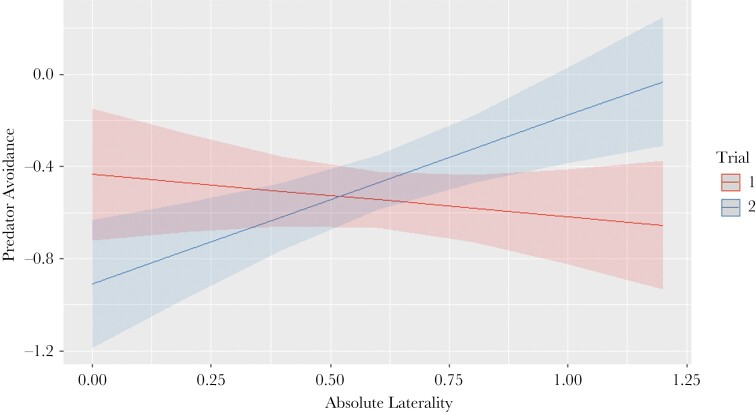
Predicted fitted line of the covariation between Absolute Laterality and Predator Avoidance, split by Trial. There was a strong, positive covariation only in the second trial.

The multivariate analysis showed no covariance between laterality and personality traits (95% CI intervals overlapping 0) but for Absolute Laterality and Predator Avoidance. While there was a positive covariance among individuals (posterior estimate: 0.008, 95% CI: 0.002–0.014), there was no effect within individuals (posterior estimate: <0.001, 95% CI: −0.006–0.007). There was no effect of treatment or trial on the covariance between the Laterality Index and any personality trait; the best-fit model, carrying 57.6% of the cumulative model weight, included no parameter other than the intercept (see Supplementary Material). There was no effect of treatment on the covariance between Absolute Laterality and any personality trait, however, the best-fit model, carrying 59.1% of the cumulative model weight, included only trial, Predator Avoidance, and the interaction between them as covariates (see Supplementary Material) (Table 3). A post-hoc analysis showed that the slopes of the covariance between Absolute Laterality and Predator Avoidance differed between trials, with such a relationship being present only in the second trial (Figure 5) (Table 4).

**Table 3:** Best fitting model after comparing AICs of different combinations of interaction effects on Absolute Laterality

Best fitting model				
Predictors	Estimate	CI		*P*
(Intercept)	−0.43	−0.72	−0.15	0.003
Trial	−0.18	−0.61	0.24	0.398
Predator Avoidance	−0.47	−0.86	−0.09	0.015
Trial × Predator Avoidance	0.91	0.32	1.51	0.003

Random effects	Estimated variance		95% CI	
Fish ID	0.02		<0.01	0.10
Cage ID	0.01		<0.01	0.05
Pond ID	0.02		<0.01	0.06

**Table 4:** Post-hoc analysis on the covariation between Absolute Laterality and Predator Avoidance, split by trial. The two LMMs have the same random effect structure as the one in Table 3, excluding fish ID.

Covariation between Absolute Laterality and Predator Avoidance								
	First trial				Second trial			
Predictors	Estimate	CI		P	Estimate	CI		*P*
(Intercept)	−0.40	−0.72	−0.08	0.015	−0.90	−1.16	−0.64	**<0.001**
Predator Avoidance	−0.24	−0.70	0.21	0.295	0.71	0.31	1.10	**<0.001**

Random effects	Estimated variance		95% CI		Estimated variance		95% CI	
Cage ID	0.04		<0.01	0.14	0.05		<0.01	0.11
Pond ID	0.04		<0.01	0.14	<0.01		<0.01	0.05

## DISCUSSION

The main aim of this study was to investigate the potential link between lateralization of behavior and personality traits as both may be involved in emotional reactions. To this end, we manipulated the environment during development to examine the effects on both laterality and personality as well as their covariation. If emotional reactions are lateralized in sticklebacks, we expected personality scores to covary with either the direction or the strength of laterality, regardless of the treatment experienced by the individual. Contrary to this, we found that only Absolute Laterality and Predator Avoidance were linked but only significantly so in the second trial. We also analyzed the direction and strength of laterality and personality traits themselves soon after we stopped the long-term manipulation of both predation threat and social density. Even though the fish did not show individual consistency in neither direction nor strength of laterality across the two trials, their lateralized behavior within each trial clearly deviated from chance. The only personality trait found to be repeatable was Activity. Although manipulation of both predation and social density during development affected growth of the fish, only the strength of laterality was influenced by predation, while the social group size influenced both Predator Avoidance and Social Tendency, suggesting an underlying effect of experienced competitive interactions on such behaviors. More precisely, the latter changed between the first and second trial only in the large group size treatment fish, while not changing in the small group size treatment.

The fact that both social group size and predation affected the size of the fish suggests that the treatments influenced the fish. The study confirmed earlier findings (Ab Ghani et al. 2016) that group size has a negative impact on body length. This could be due to the costs of living in highly social environments and increased competition for food since we provided the same amount of food *per capita*. Our study also supported the idea that predation has a positive effect on size, with fish growing faster in the presence of predator cues. This could be an adaptive response for a more efficient escape (Ab Ghani et al. 2016), or because larger fish are harder to catch (Frommen et al. 2011). Notably, the effects of group size and predation were independent, consistent with previous research (Ab Ghani et al. 2016).

We found no relationship between the direction of laterality in the mirror test and any personality trait, a result which differs from previous studies (Brown and Bibost 2014). The strength of laterality correlated positively with Predator Avoidance. This correlation was apparent only in the second trial and was independent from treatment. The relationship with Predator Avoidance is similar to previous results about the relationship between laterality and risk-taking behaviors, where it was found that lateralized individuals tended to be more shy than non-lateralized ones (Reddon and Hurd 2009; Brown and Bibost 2014). Why the relationship was only with the strength of laterality and not with direction may be a consequence of the lack of any population-level side preference: individuals in this population process emotionally relevant stimuli with either one hemisphere or the other, and the strength of this asymmetry influences the reaction to threatening stimuli of the fish. The influence of trial in this relationship is hard to explain, but it might share similarities with previous results. In convict cichlids (*Archocentrus nigrofasciatus*), it was found that risk-taking tendency was negatively related to the strength of laterality only in a familiar environment, while such covariation disappeared under novel conditions (Reddon and Hurd 2009). This might support the idea that the relationship between boldness and laterality is influenced by the novelty of the context, which was reduced in the second trial. More studies are needed to assess whether this is a consequence of stress or some other unknown factor influencing the expression of lateralized behavior.

Our population showed a bell-shaped distribution of the Laterality Index scores during the interactions with the mirror (Supplementary Materials), with no population-level direction of laterality. Moreover, there was no consistency in the direction between the two trials of the mirror test, and we have no indication of any effect of treatment on the direction of laterality. The result of the binomial analysis made for both trials suggests that more individual fish than expected by chance showed a lateralized behavior while interacting with the mirror, even though the direction of such bias was not consistent between the trials. While the strength of laterality was not consistent at the individual level across the two trials either, on average, the fish that were not exposed to predation increased their strength of laterality between the first and second trial, matching the level of the predation treatment fish. A possible explanation for the effect in the first trial is that many organisms developing in stressful conditions develop enhanced morphological asymmetries relative to control individuals (Graham et al. 2010; Frommen et al. 2011). If the predation cues made the environment more stressful for the treatment group, they could have developed a more asymmetrical body, leading to a more pronounced directional bias in behavior. However, this explanation seems unlikely because of its very short-term effect as the effect disappeared in the second trial where control fish became equally strongly lateralized. Another explanation might be that an environment with predation cues speeds up the development of laterality. Laterality enhancement allows fish to focus on multiple tasks at the same time (hence keeping attention to a threatening environment) with better efficiency (Dadda and Bisazza 2006b). The fact that such difference disappeared in the second trial by the low predation fish reaching a similar level as the high predation group indeed suggests that exposure to predation during the single trial sped up the development. This is in line with the idea that predation is an important environmental factor for the development of laterality since the water had alarm cues of damaged conspecifics during the mirror test. Previous research found that laterality can be highly plastic, especially in response to predatory cues in the environment. Studies on wild reef fish (*Pomacentrus chrysurus)* found that their strength of laterality changed over the course of 4 days, in reaction to a change in predation risk (Ferrari et al. 2015). Still, the possibility that 3 months of exposure to predator cues might be matched by a one-time exposure raises questions. It has been shown in poecilid fish that laterality is a stable trait over time (Dadda et al. 2012), with some genetic component (Bisazza et al. 2007). On the other hand, the specific eye used while interacting with stimuli seems to be very much dependent on the experience of the individual (Brown et al. 2007). More studies about the long-term effects of environmental factors, especially environmental stressors, on laterality are needed to investigate the relationship between plasticity and development and assess the effects on fitness.

Activity was consistent over time (*R* = 0.301). On the other hand, both Social Tendency (sociality) and Predator Avoidance (boldness) were not consistent traits at the individual level in our population. Due to the lack of consistency over the two trials, we cannot conclude that these individuals show personalities related to sociability or boldness (Stamps and Groothuis 2010). We are confident that the choice of the tests was adequate for our goals, since a very similar setup successfully identified consistent behavior in wild three-spined sticklebacks (Ramesh et al. 2021), and we see consistent behavior during the Activity Test. The lack of consistency in some of the tests could be related to the young age of the fish. Previous studies have found more plastic and less predictable behavior in young, sexually immature fish than in adult fish (Polverino et al. 2016). Regarding the effect of treatment on these traits, we found no effect on Activity. Predator Avoidance was reduced in fish from large groups, and the same treatment groups showed a clear reduction in their Social Tendency during the second trial. This might be explained by an average difference in stress reaction between the group size treatments. Being subject to or recovering from stressors in a social environment can alleviate stress responses compared to isolation in many different species, a phenomenon called social buffering (Gilmour and Bard 2022), that has been showed to happen in sticklebacks (Mommer and Bell 2013). If such difference can also be found between relatively small and large groups, the effect of being in a larger social group might have helped the individuals to cope with a stressful experience such as that of testing, reducing the seeking of conspecifics for safety during both the Social Preference Test (real conspecifics) and the Predator Interaction Test (mirror image in the safe area).

Ultimately, we aimed to investigate whether early life conditions affect the relationship between laterality and personality during ontogeny. Our treatment influenced laterality and social behavior, while we found the relationship between the strength of laterality and risk-taking behavior being independent of treatment. The findings suggest that there might be an underlying lateralization of stress reaction in sticklebacks, as the relationship between laterality and risk-taking behavior remained consistent across different rearing conditions. However, the study also revealed that the relationship between laterality and personality traits was highly dependent on the trial, possibly due to the novelty of the testing environment for the fish (Reddon and Hurd 2009). The lack of repeatability in both personality traits and laterality raises questions about the true nature of these measurements in the study. Nevertheless, we found that lateralized behavior in the mirror test was not random, indicating individual preferences for a particular side within each trial. It could be that our fish tended to randomly choose a turning direction while interacting with the mirror and then mostly stick to that turning “choice” during the whole test. This would generate lateralized, yet not consistent across trials, behavior. The relationship between personality and laterality might have been a consequence of such stereotypical behavior because stress-prone individuals would show a more pronounced stereotypical behavior, and they would be less likely to engage with the model predator. If this hypothesis holds true, however, such relationship should be consistently seen across trials, independent of the specific trial conditions. Moreover, one could expect this to be more apparent in the first trial, when the conditions can be considered more novel, hence more stressful. Finally, this interpretation does not explain the way lateralized behavior was influenced by the predation treatment. The data showed weaker laterality in predator-naïve individuals during the first trial, a condition one could describe as the most novel, hence most stressful for the individuals. A better explanation of the results might be that the two trials of the test were different from the individual’s perspective. Given the apparent high plasticity of behavior, individuals changed their behavior without showing consistency. However, the familiarity of the environment led to the underlying relationship between stress reaction and lateralized behavior being apparent. To confirm this hypothesis, specifically manipulating the novelty of the testing environment would be necessary to possibly avoid time effects. Future studies might investigate such relationships in older life stages when individuals have shown to behave more consistently.

## Supplementary Material

arae012_suppl_Supplementary_Material

arae012_suppl_Supplementary_Data

## Data Availability

Analyses reported in this article can be reproduced using the data provided by Paolo Panizzon Panizzon et al. (2023).
