# Supplementary material for: Effects of early predation and social cues on the relationship between laterality and personality
Source: Behav Ecol. 2024 Mar 6;35(3):arae012. doi: 10.1093/beheco/arae012 (PMC10972617; doi:10.1093/beheco/arae012)

**SUPPLEMENTARY MATERIALS IMAGES**


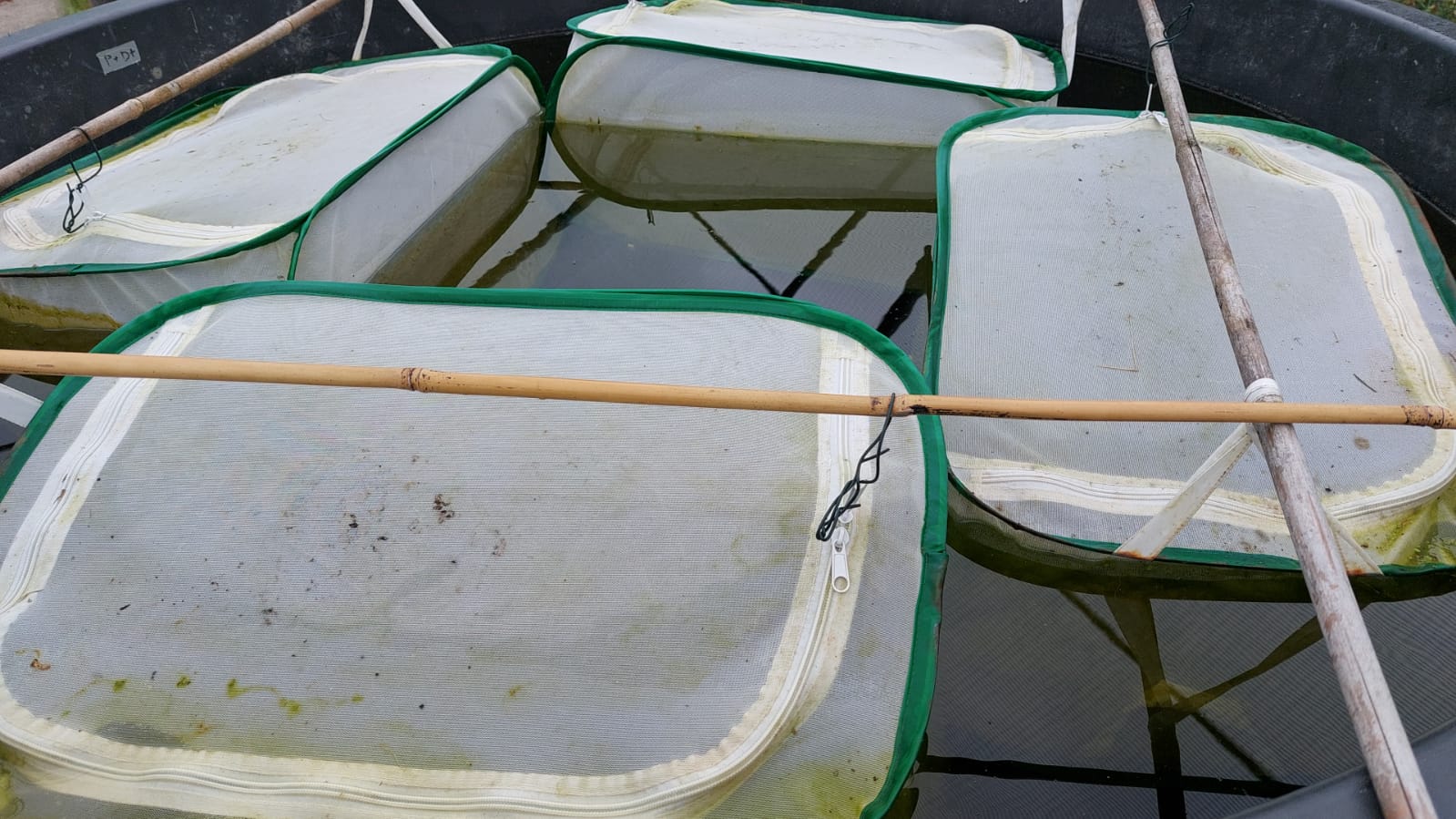


**Figure A:** Multiple cages were fit inside each pond to house the subjects.


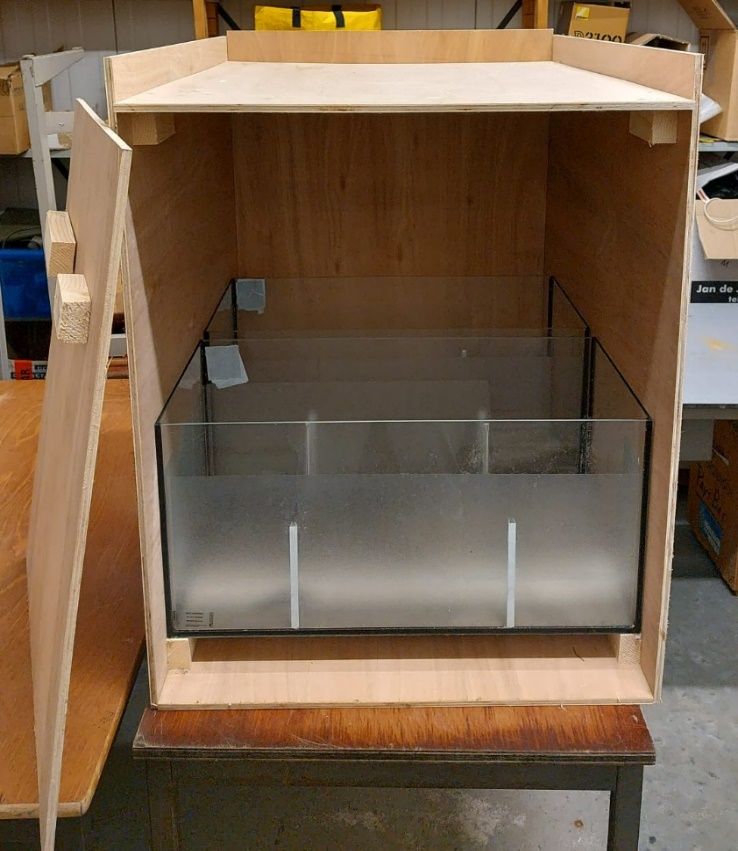


**Figure B:** Testing box with tanks inside.


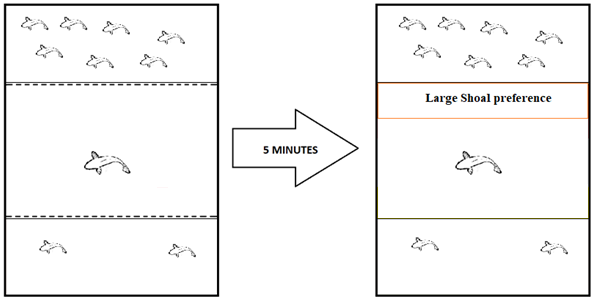


**Figure C:** Setup of the Activity/Social Preference tests.


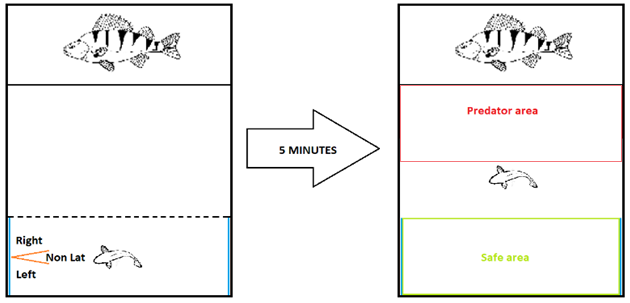


**Figure D:** Setup of the Mirror/Predator Interaction Tests.

**Figure E**: EMM of predation and group size treatments. a) Individuals from the large group size treatments are significantly smaller than those from the small group size treatment. b) Fish from the predation treatment are larger than those from the control. 95% CI and sample sizes are shown.


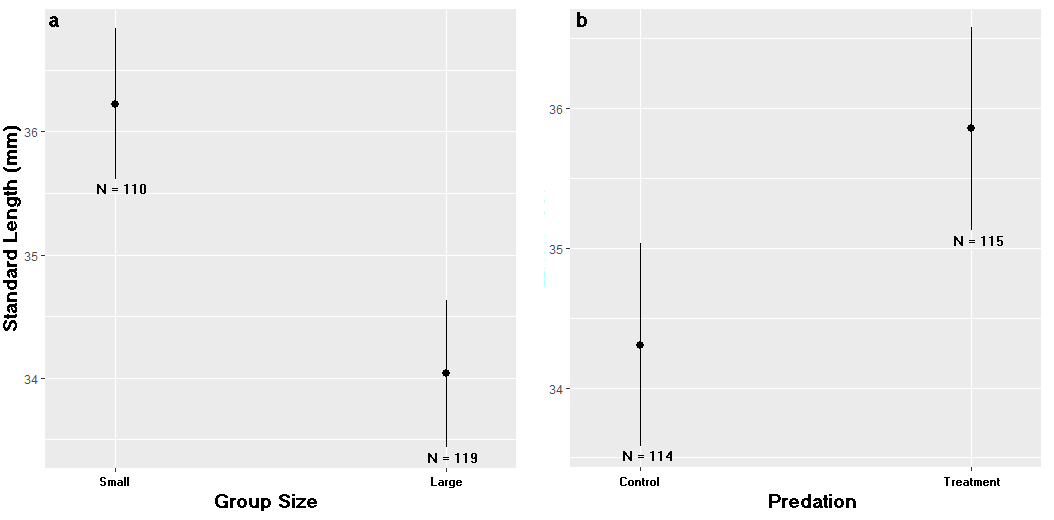

Supplement: arae012_suppl_Supplementary_Material [file arae012_suppl_supplementary_material.docx]
