# Supplementary material for: Effects of early predation and social cues on the relationship between laterality and personality
Source: Behav Ecol. 2024 Mar 6;35(3):arae012. doi: 10.1093/beheco/arae012 (PMC10972617; doi:10.1093/beheco/arae012)

**SUPPLEMENTARY MATERIALS**

**Muscle and skin extract protocol**

The extract of muscle and skin is a commonly used alarm cue for fish (Wisenden, 2015). It was obtained as follows: the heads, tails, fins, and guts of frozen dead sticklebacks were removed; then, the bodies were blended for 1 minute in tap water (600 ml per 200 g of dead individuals), and the resulting paste was then passed through filtering paper twice, to remove solid particles. The resulting liquid was stored in a freezer at -20°C.

**Tagging and measuring protocol**

Directly before the measuring and tagging procedure, fish were anesthetized in a sodium bicarbonate buffered bath of Tricaine Methane sulfonate (MS-222; 0.3 g/l; pH ~ 7.0). We determined Standard Length (straight distance between the tip of the mouth and the base of the tailfin rays) using a caliper (±0.1 mm), next we inserted a PIT tag (Passive Integrated Transponder, with each tag having a unique alphanumeric code for individual identification). To this end we made a small incision in the abdominal cavity and inserted the tag (Diameter 1.4 mm; Length 8 mm HDX PIT-tags encapsulated in biocompatible glass; Trovan). The wound was disinfected, and the fish were allowed to recover in tanks with tap water and supplied with air stones. The whole procedure lasted about 30s, and no fish needed more than 3 minutes to fully recover from the anesthesia.

**Mortality during Treatment**

Each fish originally put in the cages was given a binomial index, either 1 (survived) or 0 (dead), before tagging.

Generalized linear mixed model fit by maximum likelihood (Laplace Approximation) ['glmerMod']

Family: binomial ( logit )

Formula: SURV ~ PRED * DENS + (1 | CAGE)

Data: d

AIC BIC logLik deviance df.resid

312.4 333.2 -151.2 302.4 475

Scaled residuals:

Min 1Q Median 3Q Max

-4.1690 0.2396 0.3135 0.3151 0.4747

Random effects:

Groups Name Variance Std.Dev.

CAGE (Intercept) 0.321 0.5666

Number of obs: 480, groups: CAGE, 48

Fixed effects:

Estimate Std. Error z value Pr(>|z|)

(Intercept) 3.0891 0.6352 4.863 1.15e-06 ***

PRED1 -0.3183 0.8290 -0.384 0.701

DENS1 -0.8794 0.6881 -1.278 0.201

PRED1:DENS1 0.3043 0.9278 0.328 0.743

---

Signif. codes: 0 ‘***’ 0.001 ‘**’ 0.01 ‘*’ 0.05 ‘.’ 0.1 ‘ ’ 1

Correlation of Fixed Effects:

(Intr) PRED1 DENS1

PRED1 -0.737

DENS1 -0.890 0.677

PRED1:DENS1 0.655 -0.893 -0.741

**Effect of treatment on size**

There was a negative effect of large group size (LMM: estimate = -2.192, SEM = 0.328, p-value < 0.001, figure Ea), and a positive effect of predation treatment (LMM: estimate = 1.548, SEM = 0.522, p-value = 0.015, figure Eb) on standard length at 4 months of age. No interaction effect between predation and group size was found (LMM: estimate = -0.346, SEM = 0.656, p-value = 0.599)

**Three-way Interaction model for Laterality Index**

Linear mixed model fit by REML. t-tests use Satterthwaite's method ['lmerModLmerTest']

Formula: LAT_INDEX_NORM ~ TRIAL * PRED * DENS + (1 | ID) + (1 | CAGE) + (1 | POND)

Data: x

REML criterion at convergence: 474.3

AIC: 498.2876

Scaled residuals:

Min 1Q Median 3Q Max

-2.7167 -0.5788 -0.0090 0.5651 2.5451

Random effects:

Groups Name Variance Std.Dev.

ID (Intercept) 0.000000 0.00000

CAGE (Intercept) 0.003871 0.06221

POND (Intercept) 0.000000 0.00000

Residual 0.160476 0.40059

Number of obs: 438, groups: ID, 225; CAGE, 48; POND, 12

Fixed effects:

Estimate Std. Error df t value Pr(>|t|)

(Intercept) 0.079655 0.057991 130.508087 1.374 0.172

TRIAL2 -0.012350 0.078213 386.286103 -0.158 0.875

PRED1 -0.003335 0.081960 133.282488 -0.041 0.968

DENS1 -0.089352 0.080337 124.459527 -1.112 0.268

TRIAL2:PRED1 -0.095496 0.111197 388.295507 -0.859 0.391

TRIAL2:DENS1 0.036751 0.108168 385.583670 0.340 0.734

PRED1:DENS1 0.027427 0.113792 126.551528 0.241 0.810

TRIAL2:PRED1:DENS1 0.042378 0.153398 386.653015 0.276 0.782

Correlation of Fixed Effects:

(Intr) TRIAL2 PRED1 DENS1 TRIAL2:PRED1 TRIAL2:D PRED1:

TRIAL2 -0.669

PRED1 -0.707 0.474

DENS1 -0.722 0.483 0.511

TRIAL2:PRED1 0.470 -0.704 -0.666 -0.340

TRIAL2:DENS 0.484 -0.723 -0.343 -0.668 0.509

PRED1:DENS1 0.510 -0.342 -0.720 -0.706 0.479 0.472

TRIAL2:PRED1: -0.341 0.510 0.483 0.471 -0.725 -0.705 -0.667

optimizer (nloptwrap) convergence code: 0 (OK)

boundary (singular) fit: see help('isSingular')

**Two-way Interaction model for Laterality Index**

Linear mixed model fit by REML. t-tests use Satterthwaite's method ['lmerModLmerTest']

Formula: LAT_INDEX_NORM ~ TRIAL * PRED + TRIAL * DENS + PRED * DENS + (1 | ID) + (1 | CAGE) + (1 | POND)

Data: x

REML criterion at convergence: 472.5

AIC: 494.4512

Scaled residuals:

Min 1Q Median 3Q Max

-2.70675 -0.56647 -0.01326 0.55362 2.53316

Random effects:

Groups Name Variance Std.Dev.

ID (Intercept) 0.000000 0.00000

CAGE (Intercept) 0.003905 0.06249

POND (Intercept) 0.000000 0.00000

Residual 0.160098 0.40012

Number of obs: 438, groups: ID, 225; CAGE, 48; POND, 12

Fixed effects:

Estimate Std. Error df t value Pr(>|t|)

(Intercept) 0.08510 0.05449 105.58685 1.562 0.121

TRIAL2 -0.02337 0.06720 386.46128 -0.348 0.728

PRED1 -0.01423 0.07176 83.68766 -0.198 0.843

DENS1 -0.09978 0.07085 79.22269 -1.408 0.163

TRIAL2:PRED1 -0.07324 0.07651 387.49052 -0.957 0.339

TRIAL2:DENS1 0.05783 0.07659 386.23056 0.755 0.451

PRED1:DENS1 0.04837 0.08473 41.77686 0.571 0.571

Correlation of Fixed Effects:

(Intr) TRIAL2 PRED1 DENS1 TRIAL2:P TRIAL2:D

TRIAL2 -0.612

PRED1 -0.659 0.303

DENS1 -0.677 0.320 0.367

TRIAL2:PRED 0.345 -0.564 -0.523 0.003

TRIAL2:DENS 0.365 -0.596 -0.004 -0.536 -0.005

PRED1:DENS1 0.403 -0.002 -0.611 -0.596 -0.008 0.002

optimizer (nloptwrap) convergence code: 0 (OK)

boundary (singular) fit: see help('isSingular')

**Three-way Interaction model for Absolute Laterality**

Linear mixed model fit by REML. t-tests use Satterthwaite's method ['lmerModLmerTest']

Formula: ABS_LAT_TRANS ~ TRIAL * PRED * DENS + (1 | ID) + (1 | CAGE) + (1 | POND)

Data: x

REML criterion at convergence: 866.2

AIC: 890.2344

Scaled residuals:

Min 1Q Median 3Q Max

-2.70972 -0.60582 0.01567 0.58247 3.00836

Random effects:

Groups Name Variance Std.Dev.

ID (Intercept) 0.000000 0.0000

CAGE (Intercept) 0.008262 0.0909

POND (Intercept) 0.012591 0.1122

Residual 0.415232 0.6444

Number of obs: 427, groups: ID, 224; CAGE, 48; POND, 12

Fixed effects:

Estimate Std. Error df t value Pr(>|t|)

(Intercept) -0.72768 0.10471 53.95720 -6.950 4.99e-09 ***

TRIAL2 0.32426 0.12707 377.04498 2.552 0.01111 *

PRED1 0.41606 0.14895 56.90701 2.793 0.00710 **

DENS1 0.06711 0.13039 107.23794 0.515 0.60783

TRIAL2:PRED1 -0.48150 0.18212 379.29092 -2.644 0.00854 **

TRIAL2:DENS1 -0.14416 0.17565 376.32669 -0.821 0.41232

PRED1:DENS1 -0.25924 0.18480 109.49967 -1.403 0.16350

TRIAL2:PRED1:DENS1 0.31412 0.25021 377.28800 1.255 0.21011

---

Signif. codes: 0 ‘***’ 0.001 ‘**’ 0.01 ‘*’ 0.05 ‘.’ 0.1 ‘ ’ 1

Correlation of Fixed Effects:

(Intr) TRIAL2 PRED1 DENS1 TRIAL2:PRED1 TRIAL2:D PRED1:

TRIAL2 -0.616

PRED1 -0.702 0.436

DENS1 -0.650 0.492 0.457

TRIAL2:PRED1 0.429 -0.698 -0.616 -0.343

TRIAL2:DENS 0.446 -0.723 -0.315 -0.680 0.505

PRED1:DENS1 0.459 -0.348 -0.655 -0.706 0.494 0.480

TRIAL2:PRED1: -0.313 0.508 0.448 0.477 -0.728 -0.702 -0.678

optimizer (nloptwrap) convergence code: 0 (OK)

boundary (singular) fit: see help('isSingular')

**Two-way Interaction model for Absolute Laterality**

Linear mixed model fit by REML. t-tests use Satterthwaite's method ['lmerModLmerTest']

Formula: ABS_LAT_TRANS ~ TRIAL * PRED + TRIAL * DENS + PRED * DENS + (1 | ID) + (1 | CAGE) + (1 | POND)

Data: x

REML criterion at convergence: 866.9

AIC: 888.8762

Scaled residuals:

Min 1Q Median 3Q Max

-2.73258 -0.61433 0.02549 0.57467 3.06490

Random effects:

Groups Name Variance Std.Dev.

ID (Intercept) 1.523e-10 1.234e-05

CAGE (Intercept) 8.255e-03 9.086e-02

POND (Intercept) 1.296e-02 1.138e-01

Residual 4.157e-01 6.447e-01

Number of obs: 427, groups: ID, 224; CAGE, 48; POND, 12

Fixed effects:

Estimate Std. Error df t value Pr(>|t|)

(Intercept) -0.68642 0.09980 44.09024 -6.878 1.71e-08 ***

TRIAL2 0.24322 0.10950 377.15047 2.221 0.0269 *

PRED1 0.33200 0.13363 37.07500 2.485 0.0176 *

DENS1 -0.01108 0.11462 67.44777 -0.097 0.9233

TRIAL2:PRED1 -0.31501 0.12492 378.28987 -2.522 0.0121 *

TRIAL2:DENS1 0.01066 0.12511 376.95524 0.085 0.9321

PRED1:DENS1 -0.10196 0.13589 34.48565 -0.750 0.4582

---

Signif. codes: 0 ‘***’ 0.001 ‘**’ 0.01 ‘*’ 0.05 ‘.’ 0.1 ‘ ’ 1

Correlation of Fixed Effects:

(Intr) TRIAL2 PRED1 DENS1 TRIAL2:P TRIAL2:D

TRIAL2 -0.557

PRED1 -0.662 0.269

DENS1 -0.599 0.330 0.308

TRIAL2:PRED 0.309 -0.556 -0.471 0.007

TRIAL2:DENS 0.333 -0.598 -0.001 -0.551 -0.013

PRED1:DENS1 0.352 -0.005 -0.533 -0.591 0.001 0.008

optimizer (nloptwrap) convergence code: 0 (OK)

boundary (singular) fit: see help('isSingular')

**Three-way Interaction model for Activity**

Linear mixed model fit by REML. t-tests use Satterthwaite's method ['lmerModLmerTest']

Formula: MEAN_SPEED ~ TRIAL * PRED * DENS + (1 | ID) + (1 | CAGE) + (1 | POND)

Data: x

REML criterion at convergence: 1352.6

AIC: 1376.57

Scaled residuals:

Min 1Q Median 3Q Max

-2.2151 -0.6053 -0.1076 0.4624 3.9226

Random effects:

Groups Name Variance Std.Dev.

ID (Intercept) 0.21902 0.4680

CAGE (Intercept) 0.10998 0.3316

POND (Intercept) 0.03447 0.1856

Residual 1.13617 1.0659

Number of obs: 422, groups: ID, 228; CAGE, 48; POND, 12

Fixed effects:

Estimate Std. Error df t value Pr(>|t|)

(Intercept) 2.42124 0.19774 48.21905 12.244 <2e-16 ***

TRIAL2 -0.15519 0.20831 208.94877 -0.745 0.457

PRED1 -0.24052 0.27935 50.82829 -0.861 0.393

DENS1 0.12238 0.26279 80.63613 0.466 0.643

TRIAL2:PRED1 -0.08382 0.29402 205.06736 -0.285 0.776

TRIAL2:DENS1 -0.25966 0.29675 215.44357 -0.875 0.383

PRED1:DENS1 -0.21855 0.37171 77.14532 -0.588 0.558

TRIAL2:PRED1:DENS1 0.11026 0.41871 214.31313 0.263 0.793

---

Signif. codes: 0 ‘***’ 0.001 ‘**’ 0.01 ‘*’ 0.05 ‘.’ 0.1 ‘ ’ 1

Correlation of Fixed Effects:

(Intr) TRIAL2 PRED1 DENS1 TRIAL2:PRED1 TRIAL2:D PRED1:

TRIAL2 -0.515

PRED1 -0.699 0.365

DENS1 -0.642 0.388 0.449

TRIAL2:PRED1 0.365 -0.708 -0.514 -0.274

TRIAL2:DENS 0.362 -0.702 -0.257 -0.566 0.497

PRED1:DENS1 0.450 -0.274 -0.644 -0.705 0.386 0.400

TRIAL2:PRED1: -0.256 0.498 0.361 0.401 -0.702 -0.709 -0.563

**Two-way Interaction model for Activity**

Linear mixed model fit by REML. t-tests use Satterthwaite's method ['lmerModLmerTest']

Formula: MEAN_SPEED ~ TRIAL * PRED + TRIAL * DENS + PRED * DENS + (1 | ID) + (1 | CAGE) + (1 | POND)

Data: x

REML criterion at convergence: 1352.7

AIC: 1374.734

Scaled residuals:

Min 1Q Median 3Q Max

-2.2345 -0.5981 -0.1075 0.4578 3.9387

Random effects:

Groups Name Variance Std.Dev.

ID (Intercept) 0.22179 0.4709

CAGE (Intercept) 0.10992 0.3315

POND (Intercept) 0.03454 0.1858

Residual 1.13086 1.0634

Number of obs: 422, groups: ID, 228; CAGE, 48; POND, 12

Fixed effects:

Estimate Std. Error df t value Pr(>|t|)

(Intercept) 2.43463 0.19106 42.26285 12.743 4.56e-16 ***

TRIAL2 -0.18265 0.18029 211.62096 -1.013 0.312

PRED1 -0.26711 0.26046 38.76206 -1.026 0.311

DENS1 0.09464 0.24065 58.57000 0.393 0.696

TRIAL2:PRED1 -0.02937 0.20884 215.01383 -0.141 0.888

TRIAL2:DENS1 -0.20418 0.20888 215.23645 -0.978 0.329

PRED1:DENS1 -0.16344 0.30707 37.48154 -0.532 0.598

---

Signif. codes: 0 ‘***’ 0.001 ‘**’ 0.01 ‘*’ 0.05 ‘.’ 0.1 ‘ ’ 1

Correlation of Fixed Effects:

(Intr) TRIAL2 PRED1 DENS1 TRIAL2:P TRIAL2:D

TRIAL2 -0.462

PRED1 -0.673 0.229

DENS1 -0.609 0.237 0.357

TRIAL2:PRED 0.268 -0.582 -0.391 0.011

TRIAL2:DENS 0.265 -0.571 -0.001 -0.435 -0.001

PRED1:DENS1 0.383 0.008 -0.572 -0.633 -0.016 0.001

**Three-way Interaction model for Social Tendency**

Linear mixed model fit by REML. t-tests use Satterthwaite's method ['lmerModLmerTest']

Formula: PROP_LARGE_SHOAL ~ TRIAL * PRED * DENS + (1 | ID) + (1 | CAGE) + (1 | POND)

Data: x

REML criterion at convergence: -224.1

AIC: -200.1034

Scaled residuals:

Min 1Q Median 3Q Max

-3.5179 -0.6852 -0.0696 0.6491 2.2611

Random effects:

Groups Name Variance Std.Dev.

ID (Intercept) 0.0005248 0.02291

CAGE (Intercept) 0.0012573 0.03546

POND (Intercept) 0.0009515 0.03085

Residual 0.0309519 0.17593

Number of obs: 453, groups: ID, 229; CAGE, 48; POND, 12

Fixed effects:

Estimate Std. Error df t value Pr(>|t|)

(Intercept) 0.640835 0.028508 52.326403 22.479 <2e-16 ***

TRIAL2 0.033634 0.033248 219.294299 1.012 0.3128

PRED1 -0.002339 0.040506 56.223956 -0.058 0.9542

DENS1 0.069133 0.036140 97.808726 1.913 0.0587 .

TRIAL2:PRED1 -0.050842 0.047453 219.294299 -1.071 0.2852

TRIAL2:DENS1 -0.117383 0.046718 220.248287 -2.513 0.0127 *

PRED1:DENS1 -0.053184 0.051189 94.943513 -1.039 0.3015

TRIAL2:PRED1:DENS1 0.048864 0.066183 221.584207 0.738 0.4611

---

Signif. codes: 0 ‘***’ 0.001 ‘**’ 0.01 ‘*’ 0.05 ‘.’ 0.1 ‘ ’ 1

Correlation of Fixed Effects:

(Intr) TRIAL2 PRED1 DENS1 TRIAL2:PRED1 TRIAL2:D PRED1:

TRIAL2 -0.583

PRED1 -0.697 0.410

DENS1 -0.638 0.460 0.445

TRIAL2:PRED1 0.409 -0.701 -0.586 -0.322

TRIAL2:DENS 0.415 -0.712 -0.292 -0.643 0.499

PRED1:DENS1 0.448 -0.325 -0.644 -0.705 0.464 0.454

TRIAL2:PRED1: -0.293 0.502 0.420 0.454 -0.717 -0.706 -0.642

**Two-way Interaction model for Social Tendency**

Linear mixed model fit by REML. t-tests use Satterthwaite's method ['lmerModLmerTest']

Formula: PROP_LARGE_SHOAL ~ TRIAL * PRED + TRIAL * DENS + PRED * DENS + (1 | ID) + (1 | CAGE) + (1 | POND)

Data: x

REML criterion at convergence: -227.2

AIC: -205.1516

Scaled residuals:

Min 1Q Median 3Q Max

-3.5508 -0.6690 -0.0794 0.6602 2.2952

Random effects:

Groups Name Variance Std.Dev.

ID (Intercept) 0.0005643 0.02375

CAGE (Intercept) 0.0012583 0.03547

POND (Intercept) 0.0009472 0.03078

Residual 0.0308823 0.17573

Number of obs: 453, groups: ID, 229; CAGE, 48; POND, 12

Fixed effects:

Estimate Std. Error df t value Pr(>|t|)

(Intercept) 0.64701 0.02724 44.30188 23.753 < 2e-16 ***

TRIAL2 0.02130 0.02872 221.02819 0.742 0.45893

PRED1 -0.01492 0.03674 39.07523 -0.406 0.68693

DENS1 0.05702 0.03219 65.13561 1.771 0.08122 .

TRIAL2:PRED1 -0.02573 0.03304 222.68852 -0.779 0.43705

TRIAL2:DENS1 -0.09304 0.03305 222.54529 -2.815 0.00532 **

PRED1:DENS1 -0.02891 0.03924 35.23230 -0.737 0.46610

---

Signif. codes: 0 ‘***’ 0.001 ‘**’ 0.01 ‘*’ 0.05 ‘.’ 0.1 ‘ ’ 1

Correlation of Fixed Effects:

(Intr) TRIAL2 PRED1 DENS1 TRIAL2:P TRIAL2:D

TRIAL2 -0.527

PRED1 -0.661 0.254

DENS1 -0.593 0.301 0.315

TRIAL2:PRED 0.298 -0.565 -0.450 0.005

TRIAL2:DENS 0.307 -0.583 0.007 -0.511 -0.015

PRED1:DENS1 0.355 -0.003 -0.539 -0.605 0.006 0.002

**Three-way Interaction model for Predator Avoidance**

Linear mixed model fit by REML. t-tests use Satterthwaite's method ['lmerModLmerTest']

Formula: PROP_SAFE ~ TRIAL * PRED * DENS + (1 | ID) + (1 | CAGE) + (1 | POND)

Data: x

REML criterion at convergence: -45.5

AIC: -21.48787

Scaled residuals:

Min 1Q Median 3Q Max

-2.62413 -0.58569 0.08762 0.63836 1.87927

Random effects:

Groups Name Variance Std.Dev.

ID (Intercept) 0.0033017 0.05746

CAGE (Intercept) 0.0022628 0.04757

POND (Intercept) 0.0005117 0.02262

Residual 0.0426095 0.20642

Number of obs: 385, groups: ID, 222; CAGE, 48; POND, 12

Fixed effects:

Estimate Std. Error df t value Pr(>|t|)

(Intercept) 0.640993 0.035325 69.718527 18.145 <2e-16 ***

TRIAL2 0.027592 0.041787 186.400288 0.660 0.510

PRED1 0.003033 0.049994 70.782468 0.061 0.952

DENS1 -0.083860 0.048520 99.150615 -1.728 0.087 .

TRIAL2:PRED1 -0.093492 0.060291 190.974222 -1.551 0.123

TRIAL2:DENS1 -0.032833 0.060412 201.174380 -0.543 0.587

PRED1:DENS1 0.051278 0.068524 96.104587 0.748 0.456

TRIAL2:PRED1:DENS1 0.063880 0.085088 196.369969 0.751 0.454

---

Signif. codes: 0 ‘***’ 0.001 ‘**’ 0.01 ‘*’ 0.05 ‘.’ 0.1 ‘ ’ 1

Correlation of Fixed Effects:

(Intr) TRIAL2 PRED1 DENS1 TRIAL2:PRED1 TRIAL2:D PRED1:

TRIAL2 -0.624

PRED1 -0.706 0.443

DENS1 -0.677 0.453 0.478

TRIAL2:PRED1 0.432 -0.693 -0.613 -0.314

TRIAL2:DENS 0.432 -0.692 -0.307 -0.638 0.480

PRED1:DENS1 0.479 -0.322 -0.680 -0.708 0.446 0.452

TRIAL2:PRED1: -0.307 0.492 0.435 0.453 -0.709 -0.710 -0.638

**Two-way Interaction model for Predator Avoidance**

Linear mixed model fit by REML. t-tests use Satterthwaite's method ['lmerModLmerTest']

Formula: PROP_SAFE ~ TRIAL * PRED + TRIAL * DENS + PRED * DENS + (1 | ID) + (1 | CAGE) + (1 | POND)

Data: x

REML criterion at convergence: -48

AIC: -26.01583

Scaled residuals:

Min 1Q Median 3Q Max

-2.59547 -0.59843 0.08687 0.63276 1.91751

Random effects:

Groups Name Variance Std.Dev.

ID (Intercept) 0.0033032 0.05747

CAGE (Intercept) 0.0021969 0.04687

POND (Intercept) 0.0005165 0.02273

Residual 0.0425925 0.20638

Number of obs: 385, groups: ID, 222; CAGE, 48; POND, 12

Fixed effects:

Estimate Std. Error df t value Pr(>|t|)

(Intercept) 0.649099 0.033551 58.814720 19.347 <2e-16 ***

TRIAL2 0.012188 0.036378 190.822200 0.335 0.738

PRED1 -0.013240 0.044915 48.957523 -0.295 0.769

DENS1 -0.100364 0.043135 66.891203 -2.327 0.023 *

TRIAL2:PRED1 -0.061427 0.042516 197.752011 -1.445 0.150

TRIAL2:DENS1 -0.000649 0.042526 197.678371 -0.015 0.988

PRED1:DENS1 0.084136 0.052559 37.338210 1.601 0.118

---

Signif. codes: 0 ‘***’ 0.001 ‘**’ 0.01 ‘*’ 0.05 ‘.’ 0.1 ‘ ’ 1

Correlation of Fixed Effects:

(Intr) TRIAL2 PRED1 DENS1 TRIAL2:P TRIAL2:D

TRIAL2 -0.572

PRED1 -0.667 0.293

DENS1 -0.634 0.297 0.349

TRIAL2:PRED 0.321 -0.561 -0.481 0.011

TRIAL2:DENS 0.320 -0.560 0.003 -0.505 -0.047

PRED1:DENS1 0.386 -0.012 -0.579 -0.610 -0.011 -0.001

**Covariance between Activity and Predator Avoidance**

Linear mixed model fit by REML. t-tests use Satterthwaite's method ['lmerModLmerTest']

Formula: MEAN_SPEED ~ PROP_SAFE + (1 | ID) + (1 | CAGE) + (1 | POND)

Data: x

REML criterion at convergence: 1161.7

Scaled residuals:

Min 1Q Median 3Q Max

-2.0932 -0.5884 -0.1436 0.5155 3.8739

Random effects:

Groups Name Variance Std.Dev.

ID (Intercept) 0.17022 0.4126

CAGE (Intercept) 0.15390 0.3923

POND (Intercept) 0.06576 0.2564

Residual 1.21510 1.1023

Number of obs: 357, groups: ID, 213; CAGE, 48; POND, 12

Fixed effects:

Estimate Std. Error df t value Pr(>|t|)

(Intercept) 2.0806 0.2117 103.2407 9.827 <2e-16 ***

PROP_SAFE 0.1812 0.2950 350.3234 0.614 0.54

---

Signif. codes: 0 ‘***’ 0.001 ‘**’ 0.01 ‘*’ 0.05 ‘.’ 0.1 ‘ ’ 1

Correlation of Fixed Effects:

(Intr)

PROP_SAFE -0.842

**Covariance between Activity and Social Tendency**

Linear mixed model fit by REML. t-tests use Satterthwaite's method ['lmerModLmerTest']

Formula: MEAN_SPEED ~ PROP_LARGE_SHOAL + (1 | ID) + (1 | CAGE) + (1 | POND)

Data: x

REML criterion at convergence: 1360.8

Scaled residuals:

Min 1Q Median 3Q Max

-2.1031 -0.6143 -0.1117 0.5230 3.9127

Random effects:

Groups Name Variance Std.Dev.

ID (Intercept) 0.20415 0.4518

CAGE (Intercept) 0.10922 0.3305

POND (Intercept) 0.04762 0.2182

Residual 1.15920 1.0767

Number of obs: 424, groups: ID, 229; CAGE, 48; POND, 12

Fixed effects:

Estimate Std. Error df t value Pr(>|t|)

(Intercept) 1.9610 0.2245 171.7604 8.734 2.13e-15 ***

PROP_LARGE_SHOAL 0.3069 0.3137 401.2355 0.978 0.328

---

Signif. codes: 0 ‘***’ 0.001 ‘**’ 0.01 ‘*’ 0.05 ‘.’ 0.1 ‘ ’ 1

Correlation of Fixed Effects:

(Intr)

PROP_LARGE_ -0.896

**Covariance between Predator Avoidance and Social Tendency**

Linear mixed model fit by REML. t-tests use Satterthwaite's method ['lmerModLmerTest']

Formula: PROP_SAFE ~ PROP_LARGE_SHOAL + (1 | ID) + (1 | CAGE) + (1 | POND)

Data: x

REML criterion at convergence: -62.6

Scaled residuals:

Min 1Q Median 3Q Max

-2.72015 -0.55234 0.09288 0.66305 1.76777

Random effects:

Groups Name Variance Std.Dev.

ID (Intercept) 0.0034193 0.05847

CAGE (Intercept) 0.0032686 0.05717

POND (Intercept) 0.0001309 0.01144

Residual 0.0427451 0.20675

Number of obs: 383, groups: ID, 220; CAGE, 48; POND, 12

Fixed effects:

Estimate Std. Error df t value Pr(>|t|)

(Intercept) 0.605829 0.042882 250.304653 14.128 <2e-16 ***

PROP_LARGE_SHOAL -0.001782 0.062781 378.221668 -0.028 0.977

---

Signif. codes: 0 ‘***’ 0.001 ‘**’ 0.01 ‘*’ 0.05 ‘.’ 0.1 ‘ ’ 1

Correlation of Fixed Effects:

(Intr)

PROP_LARGE_ -0.942

**AIC comparison models with Absolute Laterality as response variable**

Global model call: lmer(formula = ABS_LAT_TRANS ~ PROP_SAFE * TRIAL + TRIAL * PROP_LARGE_SHOAL +

TRIAL * MEAN_SPEED + PROP_SAFE * TREATMENT + TREATMENT *

PROP_LARGE_SHOAL + TREATMENT * MEAN_SPEED + (1 | ID) + (1 |

CAGE) + (1 | POND), data = xfull, na.action = "na.fail")

---

Model selection table

(Int) MEA_SPE PRO_LAR_SHO PRO_SAF TRE TRI MEA_SPE:TRE MEA_SPE:TRI PRO_LAR_SHO:TRE PRO_LAR_SHO:TRI PRO_SAF:TRE

1045 -0.3674 -0.27930 +

1047 -0.5161 0.2113 -0.26790 +

1303 -0.5747 0.2934 -0.26160 + +

1 -0.5117

5 -0.6919 0.29700

1046 -0.2889 -0.031370 -0.28520 +

3 -0.6627 0.2363

7 -0.8399 0.2331 0.29550

1048 -0.4433 -0.033010 0.2254 -0.27350 +

1110 -0.4131 0.018930 -0.27940 + +

17 -0.5393 +

1304 -0.5052 -0.033220 0.3128 -0.26670 + +

21 -0.7222 0.29930 +

1053 -0.4411 -0.29070 + +

2 -0.4592 -0.023640

19 -0.7263 0.2788 +

1112 -0.5491 0.015790 0.2044 -0.26880 + +

6 -0.6384 -0.025820 0.30330

23 -0.9082 0.2779 0.29870 +

1183 -0.9600 0.7591 -0.26300 + + +

1055 -0.6208 0.2515 -0.28080 + +

275 -0.7531 0.3190 + +

4 -0.6151 -0.026590 0.2542

1368 -0.5952 0.015070 0.2715 -0.26370 + + +

279 -0.9672 0.3608 0.30480 + +

8 -0.7923 -0.028670 0.2519 0.30210

1439 -0.9857 0.7968 -0.26060 + + + +

1311 -0.6709 0.3235 -0.27500 + + +

1565 -0.2795 -0.53960 + + +

18 -0.4889 -0.021060 +

9 -0.5635 +

22 -0.6701 -0.023110 0.30460 +

1054 -0.3602 -0.030120 -0.29600 + +

13 -0.7622 0.30060 +

139 -1.0440 0.7311 + +

20 -0.6778 -0.023450 0.2902 +

1567 -0.4613 0.2380 -0.51230 + + +

11 -0.7403 0.2682 +

143 -1.2700 0.7582 0.31560 + +

1695 -0.8231 0.7435 -0.45760 + + + +

24 -0.8587 -0.025410 0.2898 0.30410 +

1184 -0.8969 -0.031170 0.7883 -0.26710 + + +

1056 -0.5448 -0.032230 0.2668 -0.28660 + +

15 -0.9326 0.2619 0.29740 +

276 -0.7065 -0.023500 0.3334 + +

280 -0.9205 -0.025630 0.3772 0.31070 + +

1823 -0.5027 0.2967 -0.50620 + + + +

1951 -0.8389 0.7661 -0.45540 + + + + +

82 -0.5927 0.022070 + +

1118 -0.4817 0.021430 -0.29360 + + +

86 -0.7897 0.023150 0.31820 + +

1312 -0.5990 -0.032480 0.3456 -0.28010 + + +

1440 -0.9256 -0.031270 0.8307 -0.26430 + + + +

25 -0.5933 + +

525 -0.5994 0.05454 + +

29 -0.7942 0.30250 + +

84 -0.7648 0.017560 0.2728 + +

1566 -0.2026 -0.029820 -0.54010 + + +

PRO_SAF:TRI df logLik AICc delta weight

1045 + 8 -303.383 623.2 0.00 0.591

1047 + 9 -303.515 625.6 2.38 0.179

1303 + 10 -303.339 627.4 4.17 0.074

1 5 -309.173 628.5 5.30 0.042

5 6 -308.501 629.3 6.04 0.029

1046 + 9 -305.438 629.5 6.23 0.026

3 6 -309.196 630.7 7.43 0.014

7 7 -308.539 631.4 8.21 0.010

1048 + 10 -305.512 631.7 8.51 0.008

1110 + 10 -306.137 633.0 9.76 0.004

17 6 -310.623 633.5 10.28 0.003

1304 + 11 -305.327 633.5 10.29 0.003

21 7 -309.929 634.2 10.99 0.002

1053 + 11 -306.051 635.0 11.74 0.002

2 6 -311.472 635.2 11.98 0.001

19 7 -310.443 635.3 12.01 0.001

1112 + 11 -306.298 635.5 12.23 0.001

6 7 -310.736 635.8 12.60 0.001

23 8 -309.753 636.0 12.74 0.001

1183 + 15 -302.659 636.9 13.69 0.001

1055 + 12 -306.009 637.1 13.82 0.001

275 8 -310.295 637.1 13.82 0.001

4 7 -311.413 637.2 13.95 0.001

1368 + 12 -306.143 637.3 14.09 0.001

279 9 -309.557 637.7 14.47 0.000

8 8 -310.687 637.8 14.61 0.000

1439 + 16 -302.522 638.9 15.64 0.000

1311 + 13 -305.845 638.9 15.66 0.000

1565 + 14 -305.388 640.2 16.94 0.000

18 7 -312.983 640.3 17.09 0.000

9 8 -312.062 640.6 17.36 0.000

22 8 -312.237 640.9 17.71 0.000

1054 + 12 -308.149 641.3 18.10 0.000

13 9 -311.396 641.4 18.15 0.000

139 12 -308.454 641.9 18.71 0.000

20 8 -312.745 642.0 18.72 0.000

1567 + 15 -305.406 642.4 19.19 0.000

11 9 -311.936 642.5 19.23 0.000

143 13 -307.638 642.5 19.25 0.000

1695 + 18 -302.095 642.5 19.27 0.000

24 9 -311.999 642.6 19.35 0.000

1184 + 16 -304.720 643.3 20.03 0.000

1056 + 13 -308.033 643.3 20.04 0.000

15 10 -311.301 643.3 20.09 0.000

276 9 -312.594 643.8 20.54 0.000

280 10 -311.796 644.3 21.08 0.000

1823 + 16 -305.254 644.3 21.10 0.000

1951 + 19 -301.961 644.5 21.27 0.000

82 8 -314.046 644.6 21.33 0.000

1118 + 13 -308.771 644.8 21.52 0.000

86 9 -313.159 644.9 21.67 0.000

1312 + 14 -307.859 645.1 21.89 0.000

1440 + 17 -304.580 645.2 21.99 0.000

25 9 -313.470 645.5 22.29 0.000

525 12 -310.591 646.2 22.98 0.000

29 10 -312.787 646.3 23.06 0.000

84 9 -313.900 646.4 23.15 0.000

1566 + 15 -307.497 646.6 23.37 0.000

[ reached getOption("max.print") -- omitted 129 rows ]

Models ranked by AICc(x)

Random terms (all models):

1 | ID, 1 | CAGE, 1 | POND

**AIC comparison models with Laterality Index as response variable**

Global model call: lmer(formula = LAT_INDEX_NORM ~ PROP_SAFE * TRIAL + TRIAL * PROP_LARGE_SHOAL +

TRIAL * MEAN_SPEED + PROP_SAFE * TREATMENT + TREATMENT *

PROP_LARGE_SHOAL + TREATMENT * MEAN_SPEED + (1 | ID) + (1 |

CAGE) + (1 | POND), data = xfull, na.action = "na.fail")

---

Model selection table

(Int) MEA_SPE PRO_LAR_SHO PRO_SAF TRE TRI MEA_SPE:TRE MEA_SPE:TRI PRO_LAR_SHO:TRE PRO_LAR_SHO:TRI PRO_SAF:TRE

1 0.03750

3 0.16250 -0.19500

5 0.09386 -9.276e-02

17 0.06675 +

19 0.23140 -0.24400 +

7 0.21830 -0.19460 -9.224e-02

2 0.06588 -0.012650

275 0.26300 -0.29140 + +

21 0.12530 -9.563e-02 +

23 0.28990 -0.24400 -9.562e-02 +

4 0.18560 -0.011500 -0.19070

1045 0.06314 6.088e-03 +

6 0.11850 -0.011900 -8.941e-02

18 0.10620 -0.016260 +

1047 0.22790 -0.23730 -1.647e-03 +

279 0.33060 -0.30190 -9.914e-02 + +

20 0.26810 -0.015750 -0.24190 +

8 0.23800 -0.010740 -0.19060 -8.924e-02

1303 0.26790 -0.29370 -5.553e-03 + +

9 0.09072 +

276 0.29570 -0.015500 -0.28420 + +

22 0.16040 -0.015500 -9.150e-02 +

24 0.32240 -0.014990 -0.24200 -9.159e-02 +

11 0.23050 -0.21140 +

1046 0.09841 -0.014860 7.207e-03 +

82 0.08046 -0.005593 + +

13 0.16200 -1.079e-01 +

84 0.24160 -0.003722 -0.24560 + +

1048 0.26080 -0.014370 -0.23550 -5.881e-04 +

280 0.35860 -0.014650 -0.29460 -9.486e-02 + +

25 0.12030 + +

27 0.30110 -0.26050 + +

15 0.30020 -0.21040 -1.065e-01 +

340 0.27200 -0.003007 -0.29380 + + +

1304 0.29640 -0.014050 -0.28690 -4.143e-03 + +

86 0.13520 -0.005869 -8.831e-02 + +

10 0.13040 -0.015970 +

283 0.32590 -0.29900 + + +

88 0.29590 -0.003989 -0.24540 -8.796e-02 + +

29 0.19370 -1.104e-01 + +

139 0.13830 -0.07146 + +

31 0.37350 -0.25990 -1.096e-01 + +

12 0.26280 -0.014560 -0.20560 +

1110 0.07685 -0.006108 7.979e-03 + +

525 0.23940 -2.244e-01 + +

1053 0.12790 2.943e-04 + +

1112 0.23810 -0.004241 -0.23880 1.715e-04 + +

344 0.33440 -0.003140 -0.30310 -9.138e-02 + + +

14 0.19710 -0.015180 -1.040e-01 +

1055 0.30760 -0.25200 -8.087e-03 + +

26 0.17210 -0.019520 + +

527 0.38790 -0.21000 -2.393e-01 + +

287 0.40650 -0.30850 -1.121e-01 + + +

155 0.19500 -0.09848 + + +

143 0.21600 -0.08176 -1.073e-01 + +

28 0.34860 -0.018800 -0.25710 + +

16 0.32850 -0.013790 -0.20490 -1.031e-01 +

1368 0.27590 -0.003411 -0.29500 -3.655e-03 + + +

PRO_SAF:TRI df logLik AICc delta weight

1 5 -135.942 282.1 0.00 0.576

3 6 -135.993 284.3 2.18 0.193

5 6 -136.898 286.1 3.99 0.078

17 6 -137.295 286.9 4.78 0.053

19 7 -136.797 288.0 5.88 0.030

7 7 -136.955 288.3 6.20 0.026

2 6 -138.833 289.9 7.86 0.011

275 8 -137.153 290.8 8.70 0.007

21 7 -138.223 290.8 8.73 0.007

23 8 -137.726 291.9 9.85 0.004

4 7 -138.930 292.2 10.15 0.004

1045 + 8 -138.362 293.2 11.12 0.002

6 7 -139.821 294.0 11.93 0.001

18 7 -140.004 294.4 12.30 0.001

1047 + 9 -137.948 294.5 12.41 0.001

279 9 -138.045 294.7 12.60 0.001

20 8 -139.533 295.5 13.46 0.001

8 8 -139.921 296.3 14.24 0.000

1303 + 10 -138.272 297.3 15.19 0.000

9 8 -140.726 297.9 15.85 0.000

276 9 -139.902 298.4 16.32 0.000

22 8 -140.973 298.4 16.34 0.000

24 9 -140.501 299.6 17.52 0.000

11 9 -140.573 299.7 17.66 0.000

1046 + 9 -141.144 300.9 18.80 0.000

82 8 -142.310 301.1 19.01 0.000

13 9 -141.526 301.6 19.57 0.000

84 9 -141.793 302.2 20.10 0.000

1048 + 10 -140.752 302.2 20.15 0.000

280 10 -140.838 302.4 20.32 0.000

25 9 -142.066 302.7 20.65 0.000

27 10 -141.330 303.4 21.31 0.000

15 10 -141.387 303.5 21.42 0.000

340 10 -142.144 305.0 22.94 0.000

1304 + 11 -141.093 305.1 22.98 0.000

86 9 -143.309 305.2 23.13 0.000

10 9 -143.446 305.5 23.41 0.000

283 11 -141.705 306.3 24.21 0.000

88 10 -142.796 306.3 24.24 0.000

29 10 -142.837 306.4 24.32 0.000

139 12 -140.701 306.4 24.36 0.000

31 11 -142.110 307.1 25.02 0.000

12 10 -143.365 307.5 25.38 0.000

1110 + 10 -143.503 307.7 25.65 0.000

525 12 -141.489 308.0 25.94 0.000

1053 + 11 -142.871 308.6 26.54 0.000

1112 + 11 -143.074 309.0 26.94 0.000

344 11 -143.114 309.1 27.02 0.000

14 10 -144.288 309.3 27.22 0.000

1055 + 12 -142.249 309.5 27.46 0.000

26 10 -144.571 309.9 27.79 0.000

527 13 -141.356 309.9 27.85 0.000

287 12 -142.457 310.0 27.87 0.000

155 13 -141.431 310.1 28.00 0.000

143 13 -141.508 310.2 28.15 0.000

28 11 -143.882 310.6 28.56 0.000

16 11 -144.217 311.3 29.23 0.000

1368 + 12 -143.397 311.8 29.75 0.000

[ reached getOption("max.print") -- omitted 129 rows ]

Models ranked by AICc(x)

Random terms (all models):

1 | ID, 1 | CAGE, 1 | POND

**Multivariate analysis**

prior <- list(R = list(V = diag(5), nu = 1), G = list(G1 = list(V = diag(5), nu = 1), G2 = list(V = diag(5), nu = 1), G3 = list(V = diag(5), nu = 1)))

Multivariate <- MCMCglmm(cbind(ABS_LAT_NORM, LAT_INDEX_NORM, PROP_SAFE, SPEED_NORM, PROP_LARGE_SHOAL) ~ TRIAL -1, random = ~ us(trait):ID + us(trait):CAGE + us(trait):POND, family = c("gaussian", "gaussian", "gaussian", "gaussian", "gaussian"), prior = prior, rcov = ~us(trait):units, nitt = 100000, burnin = 20000, thin = 10, data = xpol)

Iterations = 20001:99991

Thinning interval = 10

Sample size = 8000

DIC: -22.60928

G-structure: ~us(trait):ID

post.mean l-95% CI u-95% CI eff.samp

traitABS_LAT_NORM:traitABS_LAT_NORM.ID 0.0321189 0.022337 0.042699 8000

traitLAT_INDEX_NORM:traitABS_LAT_NORM.ID 0.0042152 -0.004936 0.015117 7427

traitPROP_SAFE:traitABS_LAT_NORM.ID 0.0004278 -0.005904 0.007433 7504

traitSPEED_NORM:traitABS_LAT_NORM.ID -0.0003260 -0.005656 0.005177 7704

traitPROP_LARGE_SHOAL:traitABS_LAT_NORM.ID -0.0004431 -0.006038 0.005157 7729

traitABS_LAT_NORM:traitLAT_INDEX_NORM.ID 0.0042152 -0.004936 0.015117 7427

traitLAT_INDEX_NORM:traitLAT_INDEX_NORM.ID 0.0495618 0.030388 0.069111 6739

traitPROP_SAFE:traitLAT_INDEX_NORM.ID -0.0001624 -0.008916 0.009097 8000

traitSPEED_NORM:traitLAT_INDEX_NORM.ID -0.0027329 -0.009823 0.004785 7376

traitPROP_LARGE_SHOAL:traitLAT_INDEX_NORM.ID -0.0012388 -0.008857 0.005991 6588

traitABS_LAT_NORM:traitPROP_SAFE.ID 0.0004278 -0.005904 0.007433 7504

traitLAT_INDEX_NORM:traitPROP_SAFE.ID -0.0001624 -0.008916 0.009097 8000

traitPROP_SAFE:traitPROP_SAFE.ID 0.0272410 0.019632 0.036035 7254

traitSPEED_NORM:traitPROP_SAFE.ID 0.0008277 -0.004135 0.005896 8117

traitPROP_LARGE_SHOAL:traitPROP_SAFE.ID -0.0003072 -0.005099 0.004789 8000

traitABS_LAT_NORM:traitSPEED_NORM.ID -0.0003260 -0.005656 0.005177 7704

traitLAT_INDEX_NORM:traitSPEED_NORM.ID -0.0027329 -0.009823 0.004785 7376

traitPROP_SAFE:traitSPEED_NORM.ID 0.0008277 -0.004135 0.005896 8117

traitSPEED_NORM:traitSPEED_NORM.ID 0.0210016 0.015426 0.026880 8000

traitPROP_LARGE_SHOAL:traitSPEED_NORM.ID 0.0007998 -0.003379 0.005065 8000

traitABS_LAT_NORM:traitPROP_LARGE_SHOAL.ID -0.0004431 -0.006038 0.005157 7729

traitLAT_INDEX_NORM:traitPROP_LARGE_SHOAL.ID -0.0012388 -0.008857 0.005991 6588

traitPROP_SAFE:traitPROP_LARGE_SHOAL.ID -0.0003072 -0.005099 0.004789 8000

traitSPEED_NORM:traitPROP_LARGE_SHOAL.ID 0.0007998 -0.003379 0.005065 8000

traitPROP_LARGE_SHOAL:traitPROP_LARGE_SHOAL.ID 0.0209177 0.015551 0.027367 8000

~us(trait):CAGE

post.mean l-95% CI u-95% CI eff.samp

traitABS_LAT_NORM:traitABS_LAT_NORM.CAGE 5.159e-02 0.02882 0.08094 8185

traitLAT_INDEX_NORM:traitABS_LAT_NORM.CAGE 5.400e-03 -0.01766 0.02989 8000

traitPROP_SAFE:traitABS_LAT_NORM.CAGE 1.288e-03 -0.01831 0.01932 8000

traitSPEED_NORM:traitABS_LAT_NORM.CAGE -8.377e-04 -0.01799 0.01763 8000

traitPROP_LARGE_SHOAL:traitABS_LAT_NORM.CAGE -2.977e-04 -0.01844 0.01667 8000

traitABS_LAT_NORM:traitLAT_INDEX_NORM.CAGE 5.400e-03 -0.01766 0.02989 8000

traitLAT_INDEX_NORM:traitLAT_INDEX_NORM.CAGE 7.180e-02 0.03660 0.11240 8000

traitPROP_SAFE:traitLAT_INDEX_NORM.CAGE -4.581e-04 -0.02191 0.02421 8000

traitSPEED_NORM:traitLAT_INDEX_NORM.CAGE -7.764e-04 -0.02324 0.02000 7587

traitPROP_LARGE_SHOAL:traitLAT_INDEX_NORM.CAGE 7.794e-04 -0.02122 0.02111 7961

traitABS_LAT_NORM:traitPROP_SAFE.CAGE 1.288e-03 -0.01831 0.01932 8000

traitLAT_INDEX_NORM:traitPROP_SAFE.CAGE -4.581e-04 -0.02191 0.02421 8000

traitPROP_SAFE:traitPROP_SAFE.CAGE 4.958e-02 0.02726 0.07634 7545

traitSPEED_NORM:traitPROP_SAFE.CAGE 6.587e-04 -0.01687 0.01790 7827

traitPROP_LARGE_SHOAL:traitPROP_SAFE.CAGE -5.067e-04 -0.01846 0.01648 8000

traitABS_LAT_NORM:traitSPEED_NORM.CAGE -8.377e-04 -0.01799 0.01763 8000

traitLAT_INDEX_NORM:traitSPEED_NORM.CAGE -7.764e-04 -0.02324 0.02000 7587

traitPROP_SAFE:traitSPEED_NORM.CAGE 6.587e-04 -0.01687 0.01790 7827

traitSPEED_NORM:traitSPEED_NORM.CAGE 4.463e-02 0.02461 0.06865 8000

traitPROP_LARGE_SHOAL:traitSPEED_NORM.CAGE 8.493e-05 -0.01676 0.01570 7356

traitABS_LAT_NORM:traitPROP_LARGE_SHOAL.CAGE -2.977e-04 -0.01844 0.01667 8000

traitLAT_INDEX_NORM:traitPROP_LARGE_SHOAL.CAGE 7.794e-04 -0.02122 0.02111 7961

traitPROP_SAFE:traitPROP_LARGE_SHOAL.CAGE -5.067e-04 -0.01846 0.01648 8000

traitSPEED_NORM:traitPROP_LARGE_SHOAL.CAGE 8.493e-05 -0.01676 0.01570 7356

traitPROP_LARGE_SHOAL:traitPROP_LARGE_SHOAL.CAGE 4.431e-02 0.02556 0.06788 8000

~us(trait):POND

post.mean l-95% CI u-95% CI eff.samp

traitABS_LAT_NORM:traitABS_LAT_NORM.POND 0.19591 0.04635 0.4295 8000

traitLAT_INDEX_NORM:traitABS_LAT_NORM.POND 0.05125 -0.18020 0.3491 8000

traitPROP_SAFE:traitABS_LAT_NORM.POND -0.01487 -0.21721 0.1724 8000

traitSPEED_NORM:traitABS_LAT_NORM.POND 0.01691 -0.14791 0.1963 7911

traitPROP_LARGE_SHOAL:traitABS_LAT_NORM.POND -0.01890 -0.24063 0.1841 8000

traitABS_LAT_NORM:traitLAT_INDEX_NORM.POND 0.05125 -0.18020 0.3491 8000

traitLAT_INDEX_NORM:traitLAT_INDEX_NORM.POND 0.37744 0.07520 0.8780 7094

traitPROP_SAFE:traitLAT_INDEX_NORM.POND -0.10431 -0.38337 0.1346 8117

traitSPEED_NORM:traitLAT_INDEX_NORM.POND 0.05924 -0.16939 0.3393 8715

traitPROP_LARGE_SHOAL:traitLAT_INDEX_NORM.POND -0.12593 -0.44977 0.1119 8000

traitABS_LAT_NORM:traitPROP_SAFE.POND -0.01487 -0.21721 0.1724 8000

traitLAT_INDEX_NORM:traitPROP_SAFE.POND -0.10431 -0.38337 0.1346 8117

traitPROP_SAFE:traitPROP_SAFE.POND 0.25119 0.05294 0.5640 8000

traitSPEED_NORM:traitPROP_SAFE.POND -0.01867 -0.21029 0.1642 8000

traitPROP_LARGE_SHOAL:traitPROP_SAFE.POND 0.09760 -0.11948 0.3874 8135

traitABS_LAT_NORM:traitSPEED_NORM.POND 0.01691 -0.14791 0.1963 7911

traitLAT_INDEX_NORM:traitSPEED_NORM.POND 0.05924 -0.16939 0.3393 8715

traitPROP_SAFE:traitSPEED_NORM.POND -0.01867 -0.21029 0.1642 8000

traitSPEED_NORM:traitSPEED_NORM.POND 0.18945 0.05064 0.4213 8299

traitPROP_LARGE_SHOAL:traitSPEED_NORM.POND -0.02446 -0.24950 0.1575 8000

traitABS_LAT_NORM:traitPROP_LARGE_SHOAL.POND -0.01890 -0.24063 0.1841 8000

traitLAT_INDEX_NORM:traitPROP_LARGE_SHOAL.POND -0.12593 -0.44977 0.1119 8000

traitPROP_SAFE:traitPROP_LARGE_SHOAL.POND 0.09760 -0.11948 0.3874 8135

traitSPEED_NORM:traitPROP_LARGE_SHOAL.POND -0.02446 -0.24950 0.1575 8000

traitPROP_LARGE_SHOAL:traitPROP_LARGE_SHOAL.POND 0.28263 0.05572 0.6446 7224

R-structure: ~us(trait):units

post.mean l-95% CI u-95% CI eff.samp

traitABS_LAT_NORM:traitABS_LAT_NORM.units 0.0518971 0.042030 0.061885 8000

traitLAT_INDEX_NORM:traitABS_LAT_NORM.units 0.0090943 -0.002335 0.020466 8000

traitPROP_SAFE:traitABS_LAT_NORM.units 0.0080904 0.002166 0.014448 8442

traitSPEED_NORM:traitABS_LAT_NORM.units -0.0012532 -0.005702 0.003755 8000

traitPROP_LARGE_SHOAL:traitABS_LAT_NORM.units -0.0022215 -0.007279 0.002840 8187

traitABS_LAT_NORM:traitLAT_INDEX_NORM.units 0.0090943 -0.002335 0.020466 8000

traitLAT_INDEX_NORM:traitLAT_INDEX_NORM.units 0.1391788 0.114353 0.163448 7639

traitPROP_SAFE:traitLAT_INDEX_NORM.units 0.0016079 -0.007979 0.011555 8000

traitSPEED_NORM:traitLAT_INDEX_NORM.units -0.0036361 -0.011525 0.003510 8000

traitPROP_LARGE_SHOAL:traitLAT_INDEX_NORM.units -0.0104421 -0.018892 -0.002218 7606

traitABS_LAT_NORM:traitPROP_SAFE.units 0.0080904 0.002166 0.014448 8442

traitLAT_INDEX_NORM:traitPROP_SAFE.units 0.0016079 -0.007979 0.011555 8000

traitPROP_SAFE:traitPROP_SAFE.units 0.0390239 0.032210 0.046764 8000

traitSPEED_NORM:traitPROP_SAFE.units -0.0005523 -0.004672 0.003512 8000

traitPROP_LARGE_SHOAL:traitPROP_SAFE.units 0.0001383 -0.004424 0.004341 8355

traitABS_LAT_NORM:traitSPEED_NORM.units -0.0012532 -0.005702 0.003755 8000

traitLAT_INDEX_NORM:traitSPEED_NORM.units -0.0036361 -0.011525 0.003510 8000

traitPROP_SAFE:traitSPEED_NORM.units -0.0005523 -0.004672 0.003512 8000

traitSPEED_NORM:traitSPEED_NORM.units 0.0234322 0.019233 0.028133 7745

traitPROP_LARGE_SHOAL:traitSPEED_NORM.units 0.0013452 -0.002276 0.004743 8000

traitABS_LAT_NORM:traitPROP_LARGE_SHOAL.units -0.0022215 -0.007279 0.002840 8187

traitLAT_INDEX_NORM:traitPROP_LARGE_SHOAL.units -0.0104421 -0.018892 -0.002218 7606

traitPROP_SAFE:traitPROP_LARGE_SHOAL.units 0.0001383 -0.004424 0.004341 8355

traitSPEED_NORM:traitPROP_LARGE_SHOAL.units 0.0013452 -0.002276 0.004743 8000

traitPROP_LARGE_SHOAL:traitPROP_LARGE_SHOAL.units 0.0278830 0.022844 0.033163 8000

Location effects: cbind(ABS_LAT_NORM, LAT_INDEX_NORM, PROP_SAFE, SPEED_NORM, PROP_LARGE_SHOAL) ~ TRIAL - 1

post.mean l-95% CI u-95% CI eff.samp pMCMC

TRIAL1 0.4099 0.2512 0.5752 7666 <1e-04 ***

TRIAL2 0.3699 0.2056 0.5292 7695 <1e-04 ***

---

Signif. codes: 0 ‘***’ 0.001 ‘**’ 0.01 ‘*’ 0.05 ‘.’ 0.1 ‘ ’ 1

**Laterality Index Histograms**


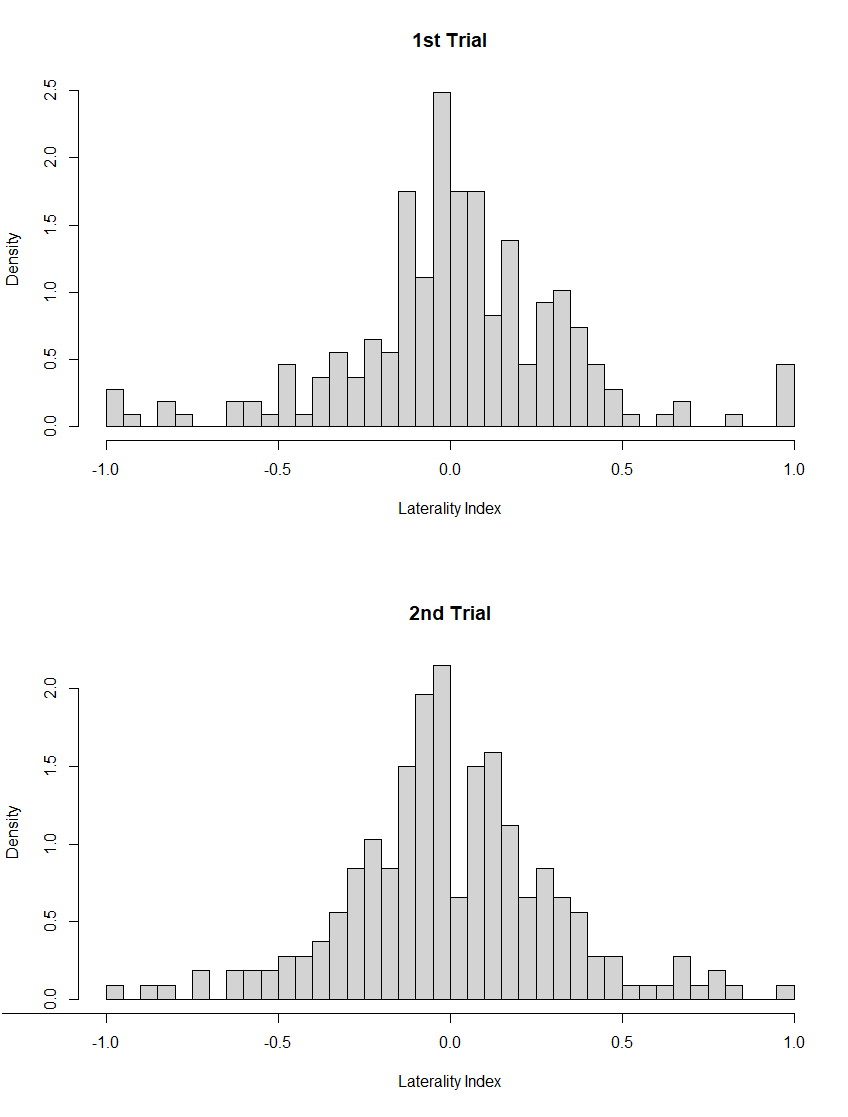

Supplement: arae012_suppl_Supplementary_Data [file arae012_suppl_supplementary_data.docx]
